# CREATE: a novel attention-based framework for efficient classification of transposable elements

**DOI:** 10.1093/bib/bbaf608

**Published:** 2025-11-16

**Authors:** Yang Qi, Yiqi Chen, Yingfu Wu, Yang Guo, Meihong Gao, Fuhao Zhang, Xingyu Liao, Xuequn Shang

**Affiliations:** School of Computer Science, Northwestern Polytechnical University, 1 Dongxiang Road, Xi’an 710129, China; School of Computer Science, Northwestern Polytechnical University, 1 Dongxiang Road, Xi’an 710129, China; School of Computer Science, Northwestern Polytechnical University, 1 Dongxiang Road, Xi’an 710129, China; School of Information Science and Engineering, Lanzhou University, 222 South Tianshui Road, Lanzhou 730000, China; School of Information, Xi’an University of Finance and Economics, 360 Changning Road, Xi’an 710100, China; College of Information Engineering, Northwest A&F University, 3 Taicheng Road, Yangling 712100, China; School of Computer Science, Northwestern Polytechnical University, 1 Dongxiang Road, Xi’an 710129, China; School of Computer Science, Northwestern Polytechnical University, 1 Dongxiang Road, Xi’an 710129, China

**Keywords:** transposable elements, hierarchical classification, deep learning, attention mechanism

## Abstract

Transposable elements (TEs) are DNA sequences that can move within a genome. They constitute a substantial portion of the eukaryotic genome and play essential roles in gene regulation and genome evolution. Accurate classification of these repetitive elements is crucial for investigating their potential impact on the genome. Over the past few decades, several alignment-based tools have been developed to annotate TE types. While these methods rely heavily on prior knowledge and are often computationally expensive, machine learning-based approaches have been proposed to overcome these limitations. However, most of these approaches fail to capture the multiscale features of TEs, resulting in suboptimal performance. Here, we propose a novel framework called CREATE, which simultaneously integrates the global pattern distribution and the local sequence profile of TEs using **C**onvolutional neural networks and **R**ecurrent neural n**E**tworks with an **A**ttention mechanism for efficient **TE** classification. Due to the hierarchical structure of TE groups, we trained nine classifiers corresponding to parent nodes within the class hierarchy. We further applied a top-down hierarchical classification strategy to achieve a more complete classification of unknown TEs. Comprehensive experiments demonstrate that CREATE outperforms existing TE-type annotation methods and achieves superior performance in hierarchical classification tasks. In conclusion, CREATE exhibits great potential for improving the accuracy of TE annotation. The source code and demo data are available at https://github.com/yangqi-cs/CREATE.

## Introduction

Transposable elements (TEs), also known as “jumping genes”, are DNA sequences capable of changing their position within the genome [1]. They are ubiquitous genetic components and are present in almost all forms of life, from prokaryotes to eukaryotes. Among these, TEs are especially abundant and structurally diverse in eukaryotic genomes, accounting for a substantial fraction of the genomic content [2]. For instance, TEs comprise $\sim $45% in humans [3] and nearly 63% in maize [4, 5]. Their prevalence and transposon features make them indispensable for understanding genome architecture and function. Extensive studies have shown that TEs play crucial roles in gene regulation, chromosomal organization, and genome evolution [6, 7]. Nevertheless, their repetitive nature poses significant challenges for sequencing, genome assembly, and annotating their structural and functional features [8]. Accurate TE classification is essential for genome annotation and provides critical insights into their roles in germline and somatic evolution [9]. However, TE classification remains difficult due to their inherent structural polymorphism and variability in sequence length.

TE classification involves categorizing TE sequences based on their transposition mechanisms and structural features. The first systematic framework was introduced by Finnegan in 1989 [10]. It divided TEs into two main classes based on their transposition intermediates. Class I elements or retrotransposons are mobilized through an RNA intermediate using a copy-and-paste mechanism. In contrast, Class II elements or DNA transposons move via a cut-and-paste mechanism. The discovery of miniature inverted-repeat transposable elements (MITEs), which are nonautonomous DNA transposons mobilized through a DNA-mediated cut-and-paste process, challenged this two-class system [11]. To address these inconsistencies, Wicker *et al.* [12] proposed a unified hierarchical classification system. This system retains the two main classes but refines their distinctions based on the presence or the absence of RNA intermediates. In this new taxonomy, Class I TEs are further subdivided into five orders: long terminal repeat (LTR), dictyostelium intermediate repeat sequence (DIRS), penelope-like elements (PLE), long interspersed nuclear element (LINE), and short interspersed nuclear element (SINE). Class II elements are divided into two subclasses. Subclass 1 elements transpose through the conventional cut-and-paste mechanism, such as terminal inverted repeats (TIRs). Subclass 2 comprises Helitrons and Mavericks, which mobilize by cleaving only one strand of donor DNA. This hierarchical system provides a foundation for accurate TE annotation and functional analysis across diverse genomes.

Based on this hierarchical TE classification system, many methods have been developed to annotate TEs in genomes. These methods can be broadly classified into three main categories: signature-based, similarity-based, and machine learning-based approaches. Signature-based approaches search for specific structural features, such as target site duplications (TSDs) and primer-binding sites, to characterize particular TE types. For example, LTR_FINDER [13] and LTR_retriever [14] are designed based on this principle to detect LTR retrotransposons, while MITE-Hunter [15] and MITEFinderII [16] are tailored to identify MITEs. However, these approaches rely on extensive prior knowledge and are limited in detecting TEs that lack clearly defined structural features [17].

Similarity-based methods identify TEs by comparing query sequences to known elements or conserved domains using search tools such as BLAST or HMMER. Dfam [18] and Repbase [19] are two widely used repeat libraries, which serve as essential resources for RepeatMasker (http://www.repeatmasker.org/) to identify genomic repeats [20]. RepeatClassifier, a TE classification module integrated in RepeatModeler, uses similarity-based strategies to classify consensus sequences by comparing genome sequences against RepeatMasker libraries and the curated TE-derived protein database [21]. Similarly, TEsorter utilizes HMMScan to search for conserved TE protein domains from databases such as GyDB [22] and REXdb [23], as described in [24]. Although widely used, similarity-based approaches are inherently limited by the comprehensiveness and quality of existing reference libraries, which hampers their ability to identify novel or divergent TEs. Furthermore, hybrid pipelines combining similarity and structure-based strategies, such as REPCLASS [25] and PASTEC [26], often require detailed prior information and sophisticated methodological frameworks.

Machine learning methods possess powerful feature learning capabilities and have been widely employed for TE classification. To handle the varying lengths of TEs, a critical first step is converting sequences into fixed-dimensional feature vectors, primarily through the $k$-mer frequency strategy. TEclass [27] is the first machine learning method utilizing support vector machines to differentiate sequences based on tetramer and pentamer frequencies. However, the $k$-mer feature may miss important sequence-specific details. RFSB, a binary random forest-based classifier that employs a selective training strategy and serves as the TE classification module in TransposonUltimate [28]. It addresses this issue by combining $k$-mer frequencies with selected protein-coding domains to construct comprehensive features, which are subsequently used to train binary classifiers for each TE type using the random forest model . Deep learning models utilize deep neural networks to automatically extract complex features and have gained widespread traction in computer vision, natural language processing, and bioinformatics. For the task of TE classification, several representative methods have been developed. DeepTE uses convolutional neural networks (CNNs) to train classifiers at each parent node in the taxonomy, and it includes a correction step based on conserved domains [29]. Transposable elements rpepresentation learner (TERL) transforms 1D sequences into 2D one-hot encoding matrices according to the longest sequence, which are then fed into CNNs for feature extraction and classification [30, 31]. Inpactor2 includes a core module, Inpactor2_Class, which performs fine-grained classification of LTR retrotransposons at the lineage level. It employs a fully connected neural network trained on $k$-mer frequency features extracted from each LTR sequence to predict the most likely lineage [32]. TEclass2 utilizes a Transformer-based architecture for TE classification. Sequences are tokenized into overlapping $k$-mers, and data augmentation techniques are applied to enhance model robustness [33]. However, its high computational demands and long training times limit practical applicability. Although these machine learning-based methods emphasize the importance of global $k$-mer distribution for classification, incorporating structural features requires extensive prior knowledge and labor-intensive sequence alignment, which always raises usability and reproducibility problems.

In addition to stand-alone classification tools, several comprehensive TE annotation pipelines incorporate classification modules as integral components of their workflows. For instance, EDTA adopts a hybrid strategy that integrates specialized signature-based predictors, such as LTR_retriever [14] for LTR retrotransposons and HelitronScanner [34] for Helitrons, and leverages RepeatModeler to detect nonstructural TEs [35]. RepeatClassifier functions not only within RepeatModeler2 but also as the core classification module in Earl Grey for TE-type annotation [36]. Moreover, REPET employs PASTEC as its dedicated classification engine [37], while FasTE integrates DeepTE to enhance classification robustness and standardization [38]. Collectively, these pipelines underscore the fact that accurate and consistent TE classification is not only a methodological requirement but also a prerequisite for constructing high-quality, nonredundant TE libraries, thereby serving as a cornerstone for reliable genome annotation.

In this study, we introduce a novel multimodule approach named CREATE, which combines CNN and a recurrent neural network (RNN) with an attention mechanism for TE classification. CREATE has several advantages: firstly, it extracts the global pattern distribution through $k$-mer frequency, improving efficiency using Horner’s rule [39, 40], and then extracts local structural features with sequences from two ends, avoiding time-consuming sequence alignment. Secondly, it employs both CNN and RNN models to learn global sequence patterns and sequential dependency characteristics effectively. Thirdly, it utilizes an attention mechanism to adaptively learn the significant features and accommodate structural variations across TEs. Finally, by training classifiers for each parent node in the hierarchical taxonomy, it supports specific TE types classification and top-down hierarchical annotation for unknown TEs [41]. Comprehensive experiments demonstrate that CREATE consistently outperforms existing deep learning-based methods across various TE classification tasks, including distinguishing TEs from non-TEs. Furthermore, compared with the state-of-the-art similarity- and machine learning-based methods, it shows superior capabilities in annotating unknown TEs and highlights its hierarchical classification performance.

## Materials and methods

**Figure 1:**
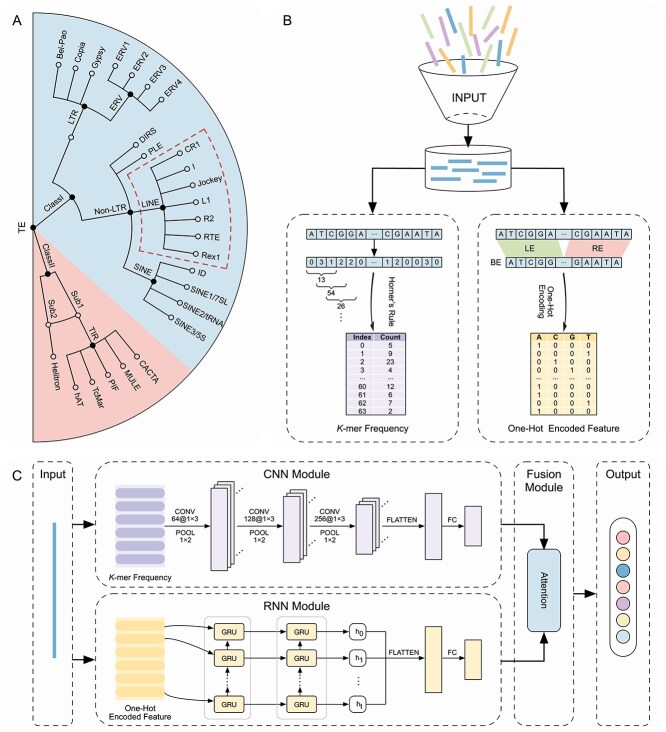
Overview of the CREATE framework. (A) Hierarchical classification structure of TEs. CREATE trains individual classifiers for the nine parent nodes (solid circles) within the hierarchical tree structure. (B) The sequence feature extraction strategy used in CREATE. Global features are captured using $k$-mer frequencies computed via Horner’s rule, while local features from both ends of sequences are transformed through one-hot encoding. LE, left end; RE, right end; BE, both ends. (C) Each classifier is implemented with an attention-based hybrid CNN–RNN architecture. CONV, convolutional layer; POOL, max-pooling layer; FC, fully connected layer; GRU, gated recurrent unit.

The CREATE framework comprises three main steps: (i) TE sequence feature extraction, in which both global and local features are extracted from each sequence; (ii) attention-based hybrid CNN–RNN model for features learning, where individual classifiers are trained for each parent node to distinguish its child nodes; (iii) hierarchical classification pipeline of TEs, which employs a top-down strategy to ensure TE-type predictions closely approximate the true annotation levels. The overall computational workflow of CREATE is illustrated in Fig. 1.

### Data collection and preprocessing

We systematically aggregated sequences from eight widely used repeat databases: CicerSpTEdb [42], DPTEdb [43], Dfam [18], MnTEdb [44], mips-REdat (PGSB repeat database) [45], Repbase (v26.02) [19], RepetDB [46], and SoyTEdb [47] (Supplementary Fig. S1). We rigorously filtered the collected dataset by removing non-TE sequences, including unannotated entries, satellite repeats, and RNA-derived sequences. The resulting data were then processed through a standardized preprocessing workflow: (i) filtering out sequences <80 bp; (ii) excluding sequences containing >20% non-canonical nucleotides; (iii) retaining unique TEs based on pairwise identity; (iv) enforcing a structured nomenclature for TEs ($ID|TE{\_}type|Species{\_}ID$). This procedure yielded 189 168 TEs, which constitute Dataset 1 for downstream tasks (Supplementary Table S1). To construct the training and test sets for each parent node, we applied a stratified sampling strategy at the leaf-node level, ensuring that sequences within each TE group were proportionally represented in both subsets. As illustrated in Supplementary Fig. S2, TEs for each leaf node were split into training (90%) and test (10%) sets, and these splits were then recursively aggregated upward to form the parent-node datasets. This approach preserves the relative composition of groups in both subsets, which is crucial given the large number of TE groups and their highly imbalanced distributions. The training sets underwent five-fold cross-validation for hyperparameter tuning and model selection, while the entirely unseen test sets were reserved to evaluate generalization performance. To further conduct a rigorous evaluation, we constructed six datasets to comprehensively assess the model’s generalizability and reliability, with their relationships and details illustrated in Supplementary Fig. S1.

**Dataset 1.** For benchmark evaluation, we used the test set from Dataset 1, which was selected through the aforementioned stratified sampling strategy (Supplementary Table S1). Hereafter, unless otherwise noted, we refer to this test set as “Dataset 1.” This dataset contains sequences annotated at the leaf-node level only, without higher-level annotations.

**Dataset 2.** Repbase is a widely used database of repetitive elements and is continuously updated. To evaluate the predictive capabilities of the trained models for future unknown sequences, we collected sequences from Repbase29.03. We then compared these with the Dataset 1 training set, which includes Repbase26.02, and used the newly added TEs in Repbase29.03 as an independent test set. After performing deduplication and standardization, 27 438 TEs remained (Supplementary Table S2). This dataset includes comprehensive leaf-node annotations and some parent-level annotations.

**Dataset 3.** Accurate annotation of active TEs is essential for investigating genome dynamics and regulatory mechanisms. To assess the ability to predict the types of active TEs, we extracted active TIR transposons from metazoans described in [48], excluding TE types that were not present in the training data, which resulted in a final test set of 86 TIR sequences. To prevent test set leakage and evaluate the method’s generalizability to novel species, we additionally extracted TIR sequences of nonmetazoans from the Dataset 1 training set, yielding 19 782 nonmetazoan TIR entries for training (Supplementary Table S3).

**Dataset 4.** To evaluate the generalization capability of the model, we used a TE database that was not included in the training set. The TREP database is a carefully curated repository of TEs annotated across multiple taxonomic ranks, including class, order, and superfamily [49]. To account for the natural heterogeneity of TEs, we selected the total_TREP dataset for hierarchical classification. After deduplication and standardization, 3333 TEs were retained to assess the performance of the hierarchical classification pipeline (Supplementary Table S2).

**Dataset 5.** TEs play a central role in adaptive evolution in eukaryotes and are especially abundant in plants [50]. Rice, as a key model organism with a small genome and rich genetic resources, is well suited for genomic studies [51]. To evaluate the accuracy of our model in predicting TE types in this model species, we used a high-quality, manually curated TE library from the EDTA consortium [35] as the reference dataset for evaluation. Following preprocessing, 2040 TE entries were retained to assess the performance of the hierarchical classification framework (Supplementary Table S2).

**Dataset 6.** To facilitate the TE classification framework and distinguish TEs from non-TEs, we collected coding sequences (CDSs) and tandem repeats (TRs) as non-TEs. TEs were derived from the original Dataset 1, including both training and test sets. Based on TE abundance statistics across Dataset 1, we selected 21 representative species and retrieved their corresponding CDS and TR sequences from NCBI (https://www.ncbi.nlm.nih.gov/datasets/genome/) and UCSC (https://genome.ucsc.edu/cgi-bin/hgGateway), respectively. After standard filtering, deduplication, and stratified sampling according to the sequence counts, we got $\sim $200 000 sequences for each non-TE type. The final dataset comprised 206 509 CDSs and 210 697 TRs, comparable to the number of TE sequences (Supplementary Table S4). The performance of this three-class classification task was evaluated using five-fold cross-validation.

### Transposable element sequence feature extraction

Encoding TE sequences into fixed-dimensional feature vectors that are suitable for model processing is challenging due to their varying lengths and complex characteristics. The $k$-mer method can extract statistical properties and the distribution of each $k$-mer motif in the whole sequence. In our study, we employ $k$-mer frequencies as global features of the TE sequence. The length of the feature is $l=4^{k}$, where $k$ represents the size of $k$-mers. To mitigate the time complexity associated with traditional sequential traversal methods, we adopt Horner’s rule to compute $k$-mer counts efficiently [39, 40]. Specifically, nucleotides A, C, G, and T are encoded as 0, 1, 2, and 3, respectively. A sliding window with $k$-length scans each sequence linearly, converting the nucleotides within the window into numerical values, which are then transformed from a quaternary representation into decimal numbers and serve as indices of the hash table (Fig. 1B). For large data, this strategy can significantly accelerate the feature extraction process. In CREATE, the preprocessed $k$-mer counts form the input layer for the CNN module. To determine the optimal $k$-mer size, we evaluated various $k$-values and selected the most suitable parameter as the final configuration.

Although $k$-mer frequencies represent potential properties of sequences, they do not include the structural patterns unique to specific TEs [52]. By considering the terminal regions of TEs that contain critical information (e.g. LTRs and TIRs), we extract features from BE of TEs and transform them using one-hot encoding. In particular, the specific length of segments is truncated from the LE and the RE of TEs, which are then concatenated into a complete BE sequence (Fig. 1B). An RNN module is employed to learn hidden features from the one-hot encoded matrices. We assessed various sequence lengths and selected the optimal length for model training.

### Attention-based hybrid convolutional and recurrent neural networks for features learning

#### Global feature learning with the convolutional neural network module

CNN is one of the most widely used deep learning models. A typical CNN consists of three types of components: convolutional layers, pooling layers, and fully connected layers. The convolutional layer, which is the core component of CNNs, applies multiple kernels to perform convolution operations on the input. Mathematically, the convolution operation is expressed as follows [53]:


\begin{align*}& x_{j}^{l} = f\left(\sum_{i\in M_{j}} x_{i}^{l-1}\ast w_{ij}^{l} + b_{j}^{l}\right) \end{align*}

$x_{j}^{l} $ denotes the $j$th channel in the layer $l$, $M_{j}$ represents a subset of the input channels, $w_{ij}^{l}$ is the convolution weight matrix, $b_{j}^{l}$ is the bias vector, and $f(\cdot )$ is the activation function.

After the convolutional layer, a pooling layer is typically applied to perform subsampling, which reduces the feature map size and mitigates overfitting. This operation can be expressed as:


\begin{align*}& x_{j}^{l}=f(pool(x_{j}^{l-1})) \end{align*}

Here, $pool(\cdot )$ denotes the subsampling function. In our CNN module, max pooling is employed.

Finally, fully connected layers are used to map the extracted features to the final output. The output of a fully connected layer is defined as:


\begin{align*}& x^{l}=f(W^{l}\cdot x^{l-1}+b^{l}) \end{align*}

where $W^{l}$ is the weight matrix and $b^{l}$ is the bias vector.

In CREATE, the CNN module comprises three convolutional blocks with 64, 128, and 256 kernels, each consisting of a convolutional layer followed by a max-pooling layer. The output of the last max-pooling layer is flattened and connected to a fully connected layer with 128 neurons (Fig. 1C). The ReLU activation function is used in all hidden layers, and a dropout rate of 0.5 is applied in the fully connected layers.

#### Local feature learning via the recurrent neural network module

To effectively capture sequential features with low computational cost, the GRU neural network is employed as the basic model for learning sequence dependencies. A general GRU network consists of two primary components: the reset gate and the update gate. The reset gate, $r_{t}$, determines how much of the previous hidden state should be ignored, while the update gate, $z_{t}$, controls how much of the previous hidden state should be carried forward to the next time step. The main GRU operations are conducted as follows [54, 55]:


\begin{gather*} z_{t} =\sigma (W_{z}x_{t}+ U_{z}h_{t-1}) \\ r_{t} =\sigma (W_{r}x_{t}+ U_{r}h_{t-1}) \\ h_{t}^{^{\prime}} =tanh(W_{h}x_{t}+ r_{t}\odot (U_{h}h_{t-1})) \\ h_{t} =(1-z_{t})\odot h_{t}^{^{\prime}}+ z_{t}\odot h_{t-1} \end{gather*}

Here, $x_{t}$ and $h_{t}$ denote the input and hidden state at time $t$. $h_{t}^{^{\prime}}$ is the candidate hidden state computed from the reset-modified previous hidden state. $\sigma (\cdot )$ and $tanh(\cdot )$ are the sigmoid and hyperbolic tangent activation functions, and $\odot $ represents elementwise multiplication.

In our study, we use two stacked GRU layers with 128 and 64 units, respectively. The output of the GRU layer is flattened, passed through a dropout layer with a rate of 0.5, and then connected to a dense layer with 128 hidden units (Fig. 1C).

#### Attention-based feature fusion module

In the CREATE framework, the CNN and RNN modules are trained in parallel to extract global and local information. An attention mechanism is then introduced to effectively fuse the outputs of these modules, allowing dynamic weight allocation to each component [56]. Specifically, the feature extracted by the CNN or RNN module, denoted as $h_{s}$, is analogous to the feature at each timestamp $s$ in a typical attention model. A fully connected layer is applied to $h_{s}$ and multiplied by a weight matrix $W$ to produce the hidden representation $e_{s}$. The normalized importance weight $\alpha _{s}$ is computed using the softmax function, and the context vector $c$ is obtained by taking the weighted sum of the features as follows:


\begin{gather*} e_{s}=tanh(W\cdot h_{s}) \\ \alpha_{s}=\frac{exp(e_{s})}{\sum_{s} exp(e_{s})} \\ c=\sum_{s}\alpha_{s}\cdot h_{s} \end{gather*}

By incorporating the attention mechanism, CREATE can automatically balance the contributions of the CNN and RNN modules, focusing on the most relevant features. During training, categorical cross-entropy is employed as the loss function, and the Adam optimizer is used with a learning rate of 0.0005.

### Hierarchical classification pipeline of transposable elements

The hierarchical structure of TEs naturally presents TE-type annotation as a hierarchical classification problem [41]. Since TE datasets exhibit substantial class imbalance due to the uneven distribution of TE types across genomes, training a single classifier across all leaf nodes may bias predictions toward the most abundant classes and compromise the recognition of rare types. To address this, we train a classifier for each parent node, thereby decomposing the overall task into smaller, manageable subproblems [57]. Combined with stratified sampling, this strategy mitigates the influence of class imbalance and enables each parent-node classifier to accurately capture distinguishing features among its child nodes.

**Figure 2:**
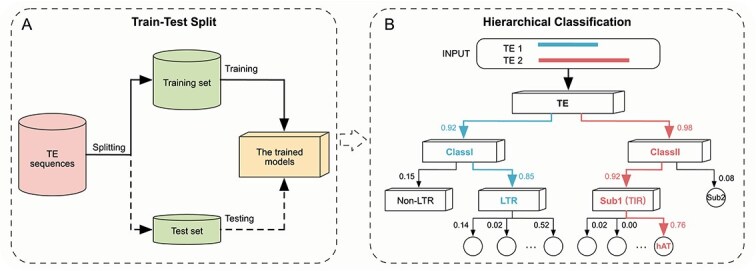
Illustration of the hierarchical classification process. (A) Preprocessed TE sequences are split into training and test sets, with dashed lines indicating the testing stage. (B) The hierarchical classification process is explained using two TE examples. Rectangular blocks represent classifiers trained for corresponding parent nodes in (A). Using a probability threshold of 0.60, TE 1 stops at LTR because the LTR model’s highest probability is 0.57, below the threshold. TE 2 continues to the leaf node hAT.

In addition, we employed a top-down hierarchical classification strategy to achieve a more comprehensive classification of unknown TEs. Given the nature of TE datasets, the classification process is treated as a nonmandatory leaf node prediction problem, allowing instances to be classified at any hierarchical level. During the testing phase, we utilized a probability threshold as a stopping criterion to determine whether an instance should be assigned to the current node or passed to the child node’s classifier [58]. If the highest confidence score predicted by the current model falls below the threshold, the process terminates at the current node, and its category is assigned as the predicted label. Conversely, the classifier proceeds to the child node with the highest predicted probability. Each sequence in the test set is recursively processed from the root node until either the stopping condition is met at a nonleaf node or the leaf node is reached (Fig. 2). This approach significantly enhances the flexibility and reliability of the model in predicting labels for unknown TEs.

### Evaluation metrics

To evaluate the classification performance of the classifiers, we used multiple classification metrics, including accuracy, precision, recall, F1-score, and Matthews correlation coefficient (MCC). These metrics are defined as follows:


\begin{gather*} Accuracy=\frac{TP+TN}{TP+FN+FP+TN} \\ Precision=\frac{TP}{TP+FP} \\ Recall=\frac{TP}{TP+FN} \\ F1-score=\frac{2\times Precision\times Recall}{Precision+ Recall} \\ MCC=\frac{TP\times TN-FP\times FN}{\sqrt{(TP+FP)(TP+FN)(TN+FP)(TN+FN)}} \end{gather*}

where TP and TN represent the number of correctly classified samples in the positive and negative classes, respectively; and FP and FN refer to the number of misclassified samples in the positive and negative categories, respectively. For benchmarking, MCC was chosen as the primary evaluation metric due to its robustness to class imbalance. Additionally, macro-averaged F1-score, precision, and recall were computed to mitigate the effect of uneven class distributions.

A key highlight of CREATE is its ability to provide an end-to-end hierarchical classification pipeline. To further evaluate the performance of this scheme, we used extended versions of the standard metrics designed for hierarchical classification, including hierarchical precision (hP), hierarchical recall (hR), and hierarchical f-measure (hF) [58, 59]. The computation method is as follows:


\begin{gather*} hP=\frac{\sum_{i} |P_{i} \cap T_{i}|}{\sum_{i} |P_{i}|}\\ hR=\frac{\sum_{i} |P_{i} \cap T_{i}|}{\sum_{i} |T_{i}|}\\ hF=\frac{2\times hP \times hR}{hP + hR} \end{gather*}

For a test sample $i$, $P_{i}$ represents the set containing the most specific predicted label along with its ancestor labels, while $T_{i}$ represents the set containing the specific true label and its ancestor labels. The hF provides a balanced evaluation by jointly considering hP and hR.

## Results

### Parameter selection and model configuration

**Figure 3:**
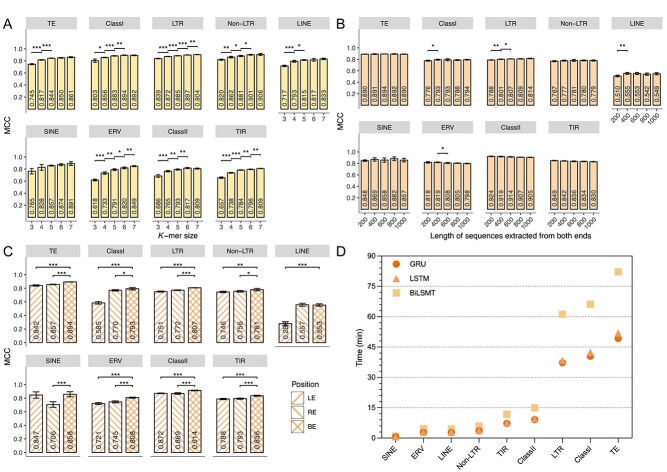
Performance comparison of different parameters and strategies in determining the architecture of CREATE. “*”, “**”, and “***” indicate significant differences with $P <.05$, $P <.01$, and $P <.001$, respectively. (A) Performance of the CNN module with different $k$-mer sizes. (B) Performance of the RNN module with different both-end sequence extraction lengths. (C) Performance of different sequence feature extraction strategies in the RNN module. (D) Training time for different RNN variants of the RNN module in CREATE.

To determine the optimal parameter and model for CREATE, we performed the five-fold cross-validation on the training set of Dataset 1. For the CNN module in CREATE, we systematically evaluated the impact of different CNN design choices, including the number of convolutional layers, kernel sizes, kernel numbers, and the structure of fully connected layers. The results indicate that these variations have minimal effect on model performance, with the exception of $k$-mer size (Supplementary Table S5). We compared $k$-mer sizes from 3 to 7 to determine the best representation for sequences. The results reveal that, except for the SINE model, increasing $k$-mer size leads to significant improvements in MCC in eight models (Fig. 3A). Although the ClassI and ClassII models yield similar results at $k=6$ and 7, $k=7$ produces the highest average performance among the other seven models. Similar improvements are observed for the F1-score, accuracy, and recall metrics (Supplementary Fig. S3). However, larger $k$-mer sizes result in sparser features, longer computation time, and greater GPU challenges. Therefore, we confined our testing of $k$-mer sizes from 3 to 7 and selected $k=7$ for subsequent experiments.

For the RNN module of CREATE, we also explored different RNN configurations, including variations in network depth and the number of GRU units per layer. Empirical results indicate that these architectural changes have minimal impact on overall performance across most TE types, with only minor differences observed in specific nodes such as LINEs (Supplementary Table S6). Considering the CNN module’s notable advantage on LINE, we focused on evaluating the RNN performance on other nodes. While deeper networks slightly increase performance for some nodes, they also substantially increase training time. Thus, by taking into account both classification accuracy and computational efficiency, we selected a two-layer GRU (128 and 64 units) with a 128-neuron fully connected layer as the final architecture, providing a balanced trade-off between performance and model complexity. Another hyperparameter is the length of the extracted BE sequences. Since the informative terminal regions of TEs typically range from tens to hundreds of base pairs, we investigated lengths from 200 to 1000 bp to achieve an optimal balance between capturing sufficient information and maintaining reasonable training times. For MCC, longer sequences do not significantly improve performance in most models, with only slight enhancement in the ClassI, LTR, and LINE models as the sequence length increased from 200 to 600 bp. Notably, the RNN module outperforms the CNN module in the ClassII and TIR models, and the performance declines with sequence length increase (Fig. 3B). Similar trends are observed for other metrics (Supplementary Fig. S4). Based on these results, we selected 600 bp as the optimal sequence length for downstream analyses.

To further validate the utility of BE sequences for TE classification, we extracted equal lengths of LE and RE sequences from TEs as input features. Guided by parameters optimized in the previous experiment, 600 bp was utilized as the sequence length. As shown in Fig. 3C (and Supplementary Fig. S5), the BE strategy consistently outperforms LE and RE methods in all models. These results emphasize the effectiveness of the BE feature extraction strategy in capturing essential structural information from TE sequences.

We also compared three RNN variants (GRU, LSTM, and BiLSTM) and the Transformer architecture to determine the optimal RNN module. The results indicate that the Transformer exhibits a notable performance decline in the LTR, ERV, and TIR models compared with other approaches. In contrast, the three RNN variants deliver comparable results across various evaluation metrics, underscoring their stability in TE classification (Supplementary Fig. S6). Furthermore, runtime comparisons on three RNN variants reveal that GRU and LSTM require similar training times for each model, and both are significantly faster than BiLSTM as the dataset size increases (Fig. 3D). Considering the simpler architecture of GRU compared with LSTM and shorter training time during five-fold cross-validation, we selected GRU as the RNN module for CREATE.

### Benchmarking performance of CREATE

**Figure 4:**
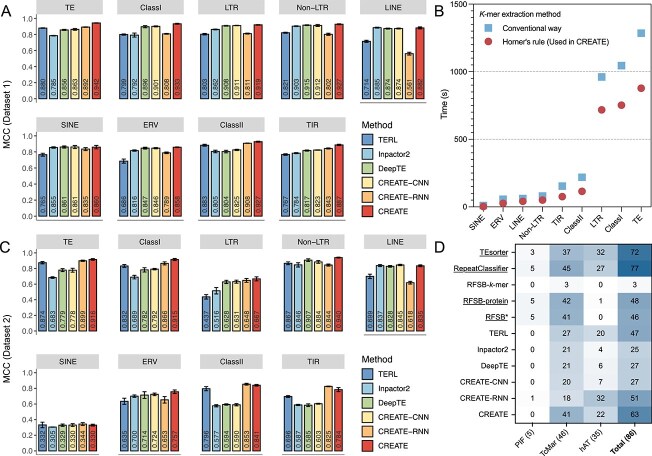
Performance comparison of different methods on parent nodes of the TE hierarchical structure. (A) MCC performance of different methods on Dataset 1. (B) Runtime comparison of $k$-mer frequency extraction between Horner’s rule and the conventional way. (C) MCC performance of different methods on Dataset 2. (D) Prediction results across various methods on Dataset 3 (86 active TIRs). Similarity-based methods are indicated by underlined names.

We benchmarked CREATE against the state-of-the-art deep learning methods TERL, Inpactor2, and DeepTE to assess performance across nine parent nodes. All methods were executed with default settings, with Inpactor2 incorporating early stopping to prevent overfitting (see Supplementary Table S7 for details). In addition, we compared the CNN–RNN fusion strategy in CREATE with models that rely only on the CNN (CREATE-CNN) or only on the RNN (CREATE-RNN) module. All methods were retrained on Dataset 1 training sets and evaluated across the test sets, with five independent runs per method. As shown in Fig. 4A, CREATE achieves the MCC consistently >0.850. It outperforms TERL by 0.044–0.172 and improves over Inpactor2 by 0.005–0.157, with a marginal decrease observed on the LINE model. Compared with DeepTE, CREATE achieves an improvement of 0.008–0.123, while showing a slightly lower score on the SINE model. Superior results are also observed across other metrics (Supplementary Figs. S7 and S8). CREATE-CNN and DeepTE employ similar CNN architectures, with slight architectural differences. Their results across nine models are almost identical, but CREATE-CNN achieves marginally better scores in five models, with improvements ranging from 0.003 to 0.021. TERL yields lower performance in seven models, which may be caused by its sequence-padding step introducing noise. Similarly, Inpactor2 underperforms CREATE-CNN in eight out of nine models. Although CREATE-RNN underperforms CREATE-CNN and DeepTE in six models, it shows significant advantages in the TE, ClassII, and TIR models, which correspond to the path involving DNA transposons in the hierarchical classification system. These findings suggest that global $k$-mer features overlook critical structural characteristics of TEs, limiting CNNs in capturing the features of DNA transposons. In contrast, local features extracted from terminal regions and processed by RNNs are better suited for this task. Previous studies have highlighted the importance of terminal repeats, particularly TIRs, in annotating DNA transposon types [12, 60]. Overall, CREATE outperforms CREATE-CNN by 0.008–0.102 (except the SINE model) and surpasses CREATE-RNN by 0.019–0.321. These results demonstrate the advantages of combining global and local features with an attention-based hybrid CNN–RNN architecture for TE classification. The confusion matrices indicate that CREATE accurately predicts various subtypes, including minority classes like Rex1 in the LINE model and SINE3/5S in the SINE model (Supplementary Fig. S9).

For the $k$-mer counts extraction process, CREATE employed Horner’s rule, which is more efficient than the traditional sequence traversal approach. As the data size grows, the time required for $k$-mer feature extraction increases significantly (Fig. 4B). When applied to all training data in Dataset 1 (the TE model), Horner’s rule improves efficiency by 32% compared with traditional methods. This strategy proves to be beneficial for processing large-scale datasets and long sequences.

### Generalization performance of CREATE

#### Parent-node classification on novel datasets

To assess the model’s generalization to previously unseen transposons, classifiers trained on the Dataset 1 training set were applied to predict TE types in Dataset 2, which contains newly added entries in Repbase29.03 compared with Repbase26.02. As shown in Fig. 4C, all methods exhibit low performance on the SINE model, with an MCC of $\sim $0.330. This reduced performance mainly arises from the severe class imbalance and the absence of certain SINE subtypes in the test set compared with the training data. Specifically, Dataset 2 contains 776 SINEs (16 SINE1/7SL, 759 SINE2/tRNA, and 1 SINE3/5S) but lacks ID-type retrotransposons. Such an imbalance leads to systematic misclassification of the missing categories, increasing false negatives and consequently lowering the MCC. Moreover, macro-averaged F1-score, precision, and recall assign equal weight to all categories (including the absent ID-type), further reducing these metrics (Supplementary Fig. S10). The presence of only one SINE3/5S sequence further amplifies this variability. In the other eight models, CREATE outperforms TERL and DeepTE, with an MCC improvement of 0.230 in the LTR model over TERL and 0.247 in the ClassII model over DeepTE. Compared with Inpactor2, CREATE shows consistent gains across seven models (except for a slight decrease on the LINE model), with the largest improvement of 0.264 observed in the ClassII model. Similarly, CREATE-RNN surpasses TERL, Inpactor2, DeepTE, and CREATE-CNN in the TE, ClassII, and TIR models. Although CREATE-RNN slightly outperforms CREATE in the ClassII and TIR models, CREATE exhibits robust and consistent performance across all models.

To evaluate both the generalizability to novel species and the ability to predict types of active TEs, we constructed a new training set by extracting TIR sequences from nonmetazoans in the Dataset 1 training set, whereas Dataset 3 comprises active TIRs from metazoans. Unlike previous evaluations across all parent nodes in Datasets 1 and 2, this experiment focuses solely on the TIR node. To benchmark performance, we included three additional methods: TEsorter (v1.4.6), RepeatClassifier (within RepeatModeler v2.0.5), and a machine learning-based binary classifier, RFSB (v1.0.0). TEsorter used the default REXdb protein-coding library for sequence alignment, while RepeatClassifier employed its default TE-encoded protein database along with the nonmetazoan training set as the reference library. RFSB was implemented in three variants: RFSB-$k$-mer, which relied solely on $k$-mer frequencies; RFSB-protein, which used only protein features; and RFSB^*^, which combined both feature types. As shown in Fig. 4D, similarity-based methods such as RepeatClassifier and TEsorter achieved the best performance, correctly classifying 77 and 72 of 86 TIRs, respectively. These are attributed to the well-defined open reading frames in TIRs and high-quality protein databases that these methods rely on. However, protein feature extraction tends to be time-consuming. The effect is most pronounced for PIF transposons. Almost all similarity-based methods recovered all five PIF sequences, whereas among machine learning-based methods, only CREATE-RNN identified one sequence. Analysis of the alignment information revealed that all five PIF sequences contain DDE superfamily endonuclease domains, specifically DDE_Tnp_4 and, in some cases, DDE_Tnp_1. Sequence alignment of these domains confirmed the characteristic DDE motif, consistent with their functional role as transposases. Furthermore, annotation review from the original literature [48] indicated varying degrees of sequence divergence, with two sequences exceeding 20%, which may partly explain the poor performance of machine learning-based methods. RFSB-protein correctly predicts 48 TIRs, outperforming RFSB-$k$-mer and further confirming the role of protein-coding domains for this classification task. Among the machine learning approaches, CREATE achieves the highest accuracy by correctly predicting 63 sequences, with notable performance in the TcMar (41/46) and hAT categories (22/35). CREATE-RNN correctly predicts 51 sequences, which highlights the advantage of the RNN module in capturing terminal structural features. These results underscore the superior performance of the RNN module within the CREATE framework for TIR classification and without alignment.

#### Hierarchical classification across multiple datasets

**Figure 5:**
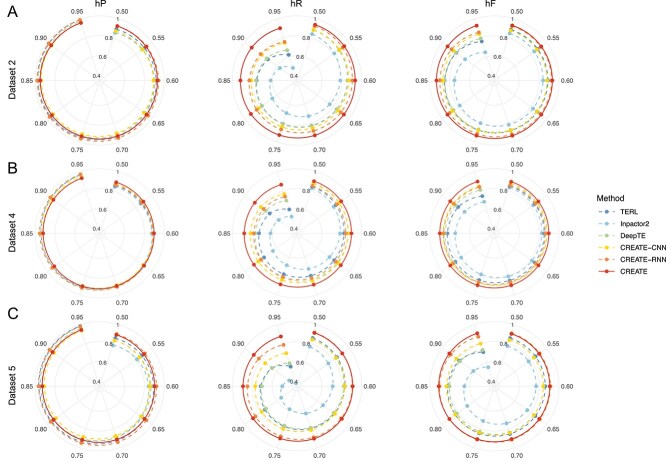
Comparison of the hierarchical classification performance of different methods. (A–C) Hierarchical classification results at different probability thresholds range from 0.50 to 0.95 for Datasets 2, 4, and 5, respectively. These datasets contain labels at various group levels, allowing for a thorough evaluation of hierarchical classification across methods.

Using the hierarchical classification strategy described previously, we evaluated CREATE on Datasets 2, 4, and 5, which provide labels at various levels, enabling comprehensive assessment of hierarchical classification performance. We compared CREATE with other methods capable of adapting to this hierarchical classification strategy, including TERL, DeepTE, CREATE-CNN, and CREATE-RNN. Starting from the root node, the prediction process could stop at any level based on the predefined threshold. The probability threshold ranged from 0.50 to 0.95 in increments of 0.05. In this scenario, a higher threshold reduces the likelihood of passing predictions to child nodes, resulting in shorter predicted label paths and higher hP scores. Conversely, a lower threshold may provide more detailed category information and produce higher hR values. The hF offers a balanced assessment by integrating hP and hR metrics. As shown in Fig. 5, only minor differences in hP are observed between the six methods. However, hR scores exhibit substantial variation across datasets, with CREATE maintaining a clear advantage. For hF, CREATE outperforms the other methods across all datasets. Overall, CREATE demonstrates remarkable robustness and insensitivity to threshold variations, achieving superior label prediction performance across all evaluation metrics.

We compared CREATE with several alternative methods to evaluate its hF performance. For deep learning-based methods (TERL, Inpactor2, DeepTE, and CREATE), we defined the best hF as the final result across various probability thresholds. DeepTE^*^ introduces the correction step based on DeepTE with conserved domains. All compared methods were executed with default parameters. RepeatClassifier utilized its default TE-encoded protein database together with Dataset 1 training sets as the reference library. As shown in Table 1, the similarity-based methods, TEsorter and RepeatClassifier, perform worse than the machine learning and deep learning-based approaches. Among the machine learning-based methods, RFSB^*^, which incorporates $k$-mers with protein-coding domains, outperforms RFSB-$k$-mer and slightly surpasses RFSB-protein, highlighting the importance of including protein information for accurate annotation of unknown TEs. TERL and DeepTE show consistently strong performance across these three datasets, outperforming RFSB^*^ and Inpactor2. Moreover, compared with the original DeepTE, the correction step implemented in DeepTE^*^ improves hF by 0.010 on Dataset 2 and by 0.001 on Dataset 5. Overall, CREATE gets the highest hF in all datasets, outperforming the best results of the comparing methods by 0.045, 0.020, and 0.049 on Datasets 2, 4, and 5, respectively.

**Table 1:** The hierarchical classification performance (hF) of different methods on Datasets 2, 4, and 5. These datasets contain labels at various group levels, allowing for a thorough evaluation of hierarchical classification across methods.

	TEsorter	RC^a^	RFSB^b^$k$	RFSB^c^	RFSB^*^	TERL	Inpactor2	DeepTE	DeepTE^*^	CREATE
Dataset 2	0.530	0.710	0.811	0.883	0.884	0.888	0.847	0.878	0.888	**0.933**
Dataset 4	0.270	0.766	0.749	0.846	0.847	0.851	0.834	0.879	0.879	**0.899**
Dataset 5	0.116	0.553	0.708	0.733	0.738	0.861	0.775	0.842	0.843	**0.910**

^a^RepeatClassifier; ^b^RFSB-*k*-mer; ^c^RFSB-protein. Bold values indicate the best results.

### Interpretability analysis through feature attribution

To interpret the predictions of CREATE, we performed feature attribution analyses using SHAP (SHapley Additive exPlanations), which estimates the contribution of each input feature to the model output [61]. Sequence feature contributions were evaluated separately for the CNN and RNN modules. Additionally, attention weights in CREATE were visualized to highlight contributions emphasized during prediction. This combined analysis provides complementary and interpretable insights into the sequence determinants underlying TE classification.

For the CNN module, which captures global $k$-mer frequency patterns, input features derived from Dataset 1 training sequences were fed into the CREATE-CNN model. To investigate individual 7-mer contributions, we applied DeepExplainer using subsets of sequences due to GPU memory limitations. Specifically, 100 sequences were randomly sampled from the training set as background, and 50 sequences from each subtype were selected from the Dataset 1 test set as test samples. This procedure was repeated five times to ensure robustness, and SHAP values were aggregated to visualize the contributions of different 7-mer features (Supplementary Fig. S11). Single-nucleotide repeats and simple repetitive sequences are preferentially retained by the $k$-mer-based feature extraction strategy, and their well-defined structures are readily observable in SHAP analyses. For the non-LTR elements, the 7-mer motif AAAAAAA exhibits strong contributions. Further examination reveals that this motif was predominantly associated with LINE elements, with L1 retrotransposons displaying a pronounced AAAAAAA signal, consistent with the A-rich 3’ end features, including poly(A) tails, TRs, or A-rich regions, reported in previous studies [12]. A/T-rich patterns are also observed within L1 sequences. Although previous studies have reported a strong A/T-rich targeting tendency in L1 elements, the internal A/T composition requires further detailed analysis [62]. It should be noted that caution is required when interpreting the contributions of other $k$-mers, as shorter or less repetitive motifs may exhibit greater variability across runs or depend on specific subsets of sequences.

For the RNN module, which models local dependencies in the terminal regions of TEs, sequence feature importance was evaluated using the CREATE-RNN model. We used 200 sequences from the training set as background and 100 sequences from each test subtype as test samples. CACTA elements, ranging from a few hundred to over 20 000 bp, display pronounced terminal motifs critical for subtype prediction (Supplementary Fig. S12A), consistent with the previously reported 5’-CACTA...TAGTG-3’ motif before a TSD [12]. While the specific motifs vary depending on the randomly selected 100 test samples, the pronounced contributions from both terminal regions of the sequences remain consistent across different runs. In SINE2/tRNA elements, clear T-rich signals are detected in the 3’ region (Supplementary Fig. S12B), aligning with reports that these sequences can be A- or AT-rich or a poly(T) tail representing the Pol III termination signal [63]. These observations support the effectiveness of the RNN module while indicating that more precise analyses require careful control of sequence quality and species context.

**Figure 6:**
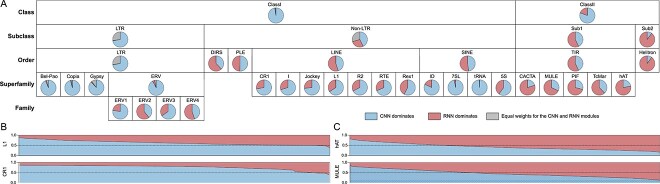
Attention weight visualization during the testing phase of Dataset 1. (A) Proportion of sequences with dominant CNN weights, dominant RNN weights, or equal attention weights from both models across different TE groups. (B and C) Distribution of CNN and RNN attention weights for the two largest superfamilies in each order: L1 and CR1 in LINE (B), and hAT and MULE in TIR (C).

We also visualized the attention weights in the fusion module to assess the relative contribution of CNN and RNN features across TE types. For Dataset 1, ClassII elements show higher RNN weights than ClassI, highlighting the advantage of the RNN module (Fig. 6A). At the subclass level, $\sim $30% LTRs and non-LTRs display a collaborative contribution from both modules. In addition, CNN features dominate in LTR predictions, and 28% of non-LTR sequences exhibit higher RNN weights. Within ClassII, Sub2 transposons show higher RNN weights, reaching 90%, due to Helitron sequences exhibiting distinct terminal features [64]. For superfamilies in LTR, nearly 90% of sequences have higher CNN weights. In non-LTR, we further analyzed the attention weight of L1s and CR1s, the most abundant subcategories in LINE, revealing that 66% and 72% of CNN weights exceed 0.5, respectively (Fig. 6B). The dominance of CNN weights in the majority of L1 subtype predictions is consistent with SHAP-based analyses, which highlight the specific motifs as dominant contributors. For superfamilies in TIR, sequences with higher RNN weights are prominent. Similarly, we visualized the weights for hAT and MULE elements, with RNN weights dominating 78% and 68% of entries, respectively (Fig. 6C). Notably, 80% of CACTA sequences show higher RNN weights in subtype prediction, consistent with SHAP-based analyses highlighting their terminal motifs as key features. These results indicate that local structural sequences from BE sequences are crucial for the composition of ClassII. This observation is consistent with Fig. 4A, where CREATE-RNN outperforms the other methods on TE, ClassII, and TIR models and further confirms that CREATE can adaptively learn the relative importance of different features, thereby enhancing overall classification performance.

### Transposable element versus non-transposable element sequences classification

Accurately distinguishing TEs from non-TEs is a critical requirement for TE annotation tools in practical applications, enabling the removal of false positives among detected repetitive sequences. Considering the application scenarios and overall performance, we compared four deep learning-based methods, TERL, Inpactor2, DeepTE, and CREATE, for TE versus non-TE classification. We performed a three-class classification (CDS, TR, and TE) using five-fold cross-validation on Dataset 6. As shown in Supplementary Fig. S13, CREATE achieves the best performance, with all metrics exceeding 0.995 in three subgroups. DeepTE achieves nearly 0.995 in the TR group, surpassing its performance in the CDS and TE groups, due to the strength of $k$-mer frequencies in characterizing the repetitive patterns of TRs. The performance of TERL varies from 0.970 to 0.990, with its highest performance in the CDS group. Inpactor2 performs worse than other methods, suggesting that the fully connected neural networks are limited in capturing sequence-specific features. Although all methods perform well for different groups, CREATE stands out with an average of 0.997, confirming its superiority in this classification task.

## Discussion

Accurate classification of TEs is essential for understanding their biological roles. However, TE classification remains challenging due to the diversity of TE types and their variable lengths. In this study, we introduce CREATE, an attention-based hybrid CNN–RNN framework for TE classification. CREATE integrates a TE-specific both-end sequence extraction strategy to capture local terminal features, complementing the traditional $k$-mer frequency approach encodes global sequence features. With an attention mechanism, it dynamically fuses global features learned by the CNN module with local features captured by the RNN module, effectively modeling the pattern distribution and structural information of TEs. Furthermore, CREATE employs a hierarchical classification pipeline to accommodate end-to-end TE annotation.

The superior performance of CREATE across diverse datasets demonstrates the benefits of its design. CREATE-CNN consistently captures global sequence features across multiple parent nodes, while CREATE-RNN excels in Class II and TIR models by effectively extracting local features from terminal sequences. By adaptively balancing global and local features, facilitated by the attention mechanism, CREATE outperforms single-module variants. Comprehensive experiments against high-quality TE datasets from diverse sources reveal that CREATE surpasses existing deep learning-based methods at each parent node and excels in classifying active DNA transposons. Benchmarking experiments on multiple public datasets demonstrate that CREATE surpasses the state-of-the-art approaches in hierarchical TE classification.

Despite these strengths, there are still limitations that should be considered in future studies. Specifically, due to the hierarchical structure of TEs and inherent class imbalance, CREATE requires training classifiers for each parent node, which increases the computational complexity for new datasets. Moreover, errors at higher-level classifications may propagate downward, potentially affecting predictions at finer-grained levels. In addition, learning-based methods remain less effective than similarity-based approaches for TE types with conserved domains but high sequence divergence, highlighting the continued value of alignment-based strategies for motif-conserved families. Future work should focus on developing an integrated TE classification framework that enhances usability and reliability while incorporating complementary approaches to improve recognition of motif-conserved families without compromising overall performance.

## Conclusion

CREATE is a novel attention-based hybrid CNN–RNN framework for TE classification. The CNN module learns global pattern distribution from $k$-mer frequencies, while the RNN module processes local sequence profiles from both ends of TEs. An attention mechanism automatically balances the contributions of these two modules. Ablation studies confirmed the effectiveness of the individual CNN and RNN modules and highlighted the advantage of the attention-based fusion strategy. Extensive benchmarking results suggest that CREATE outperforms existing methods across various datasets in hierarchical TE classification, underscoring its potential as a powerful tool for TE-type annotation. In the future, CREATE will be integrated into TE identification pipelines to facilitate genome annotation research.

**Key Points**

- We introduce a novel framework, CREATE, that classifies transposable elements (TEs) by simultaneously employing a convolutional neural network module to learn global $k$-mer frequency features and a recurrent neural network module to capture local features from both terminal regions.
- An attention mechanism automatically balances the contributions of global and local information, effectively enhancing the robustness of TE classification. The attention weights reflect the contribution of pattern distributions and structural features to TE-type annotation.
- CREATE not only excels in annotating specific TE types but also demonstrates superior performance in hierarchical TE classification tasks.

## Supplementary Material

Supplementary_bbaf608

## Data Availability

All data used in this study are publicly available and are referenced in the manuscript. The source code and demo data of CREATE are available at https://github.com/yangqi-cs/CREATE.
