# Supplementary material for: CREATE: a novel attention-based framework for efficient classification of transposable elements
Source: Brief Bioinform. 2025 Nov 16;26(6):bbaf608. doi: 10.1093/bib/bbaf608 (PMC12619909; doi:10.1093/bib/bbaf608)
Supplement: Supplementary_bbaf608 [file supplementary_bbaf608.docx]

**Supplementary Material**

**CREATE:** **a novel attention-based framework for efficient classification of transposable elements**

Qi *et al.*

**Supplementary Figures**

**
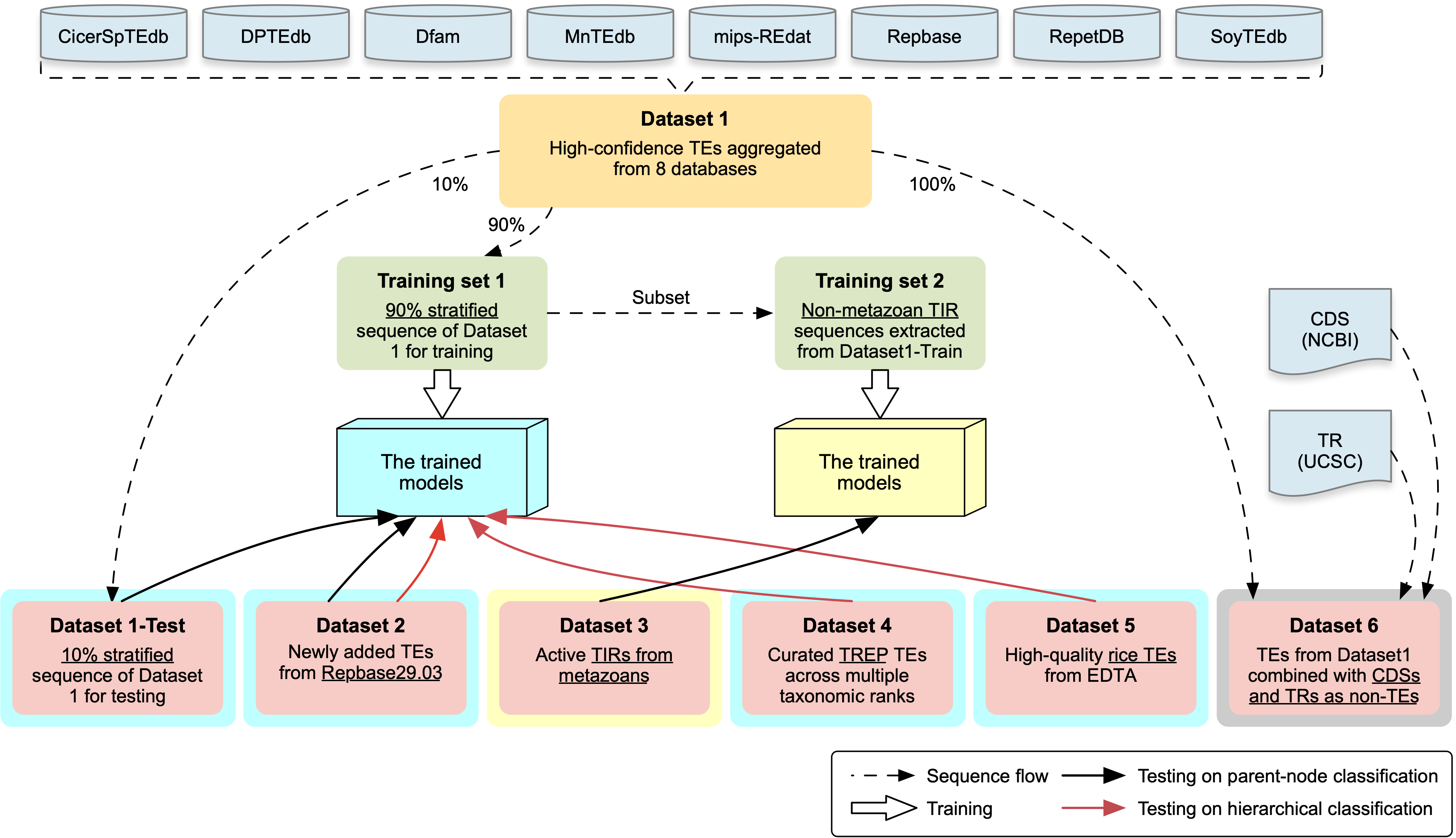
**

**Fig. S1.** Overview of the relationship between six datasets and the experimental setup for testing parent-node and hierarchical classification. Dashed arrows represent the flow of sequences between datasets. Black arrows indicate parent-node classification tests, and red arrows indicate hierarchical classification tests.


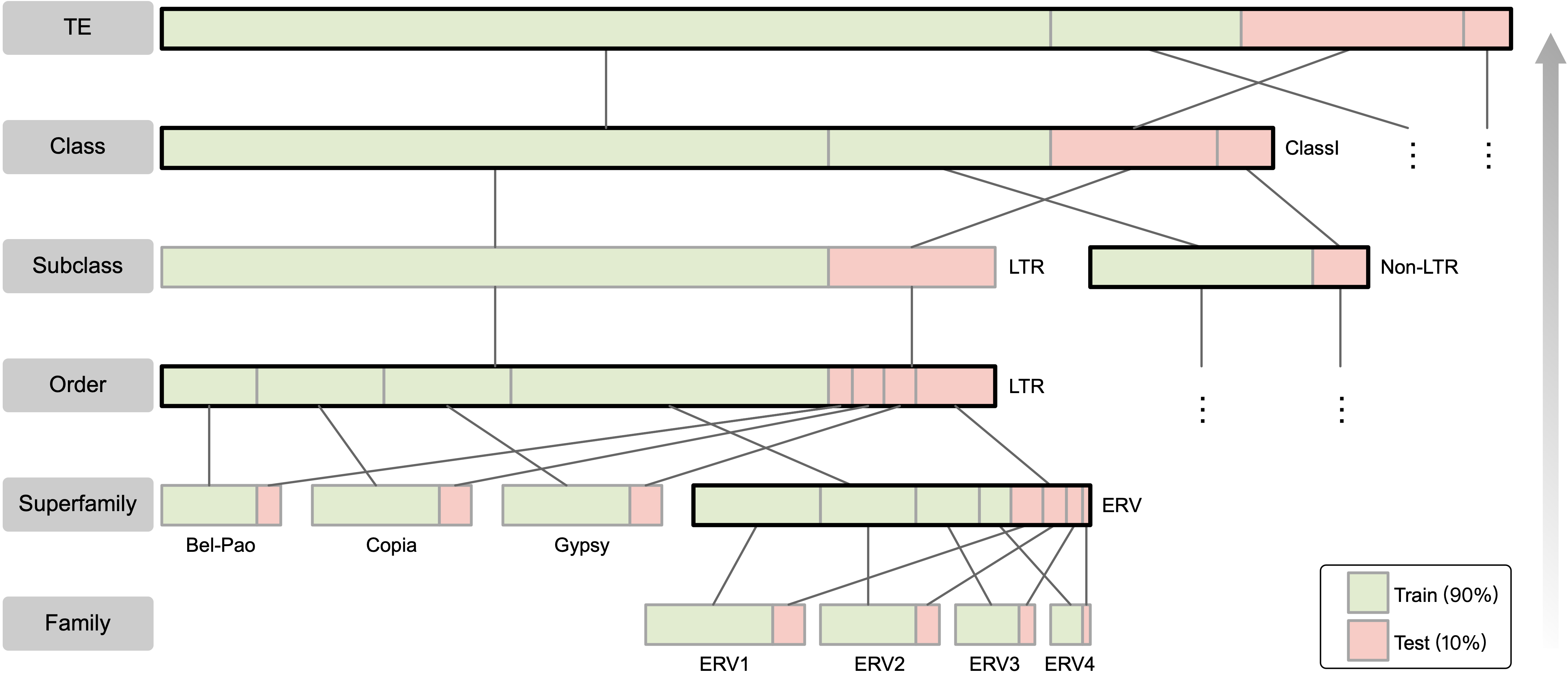


**Fig. S2.** Data splitting strategy using stratified sampling across hierarchical levels of TEs. The process proceeds bottom-up, starting with leaf nodes, where stratified sampling divides data into training (90%) and test (10%) sets. These splits are propagated upward to construct the parent node's datasets, highlighted with bold black borders. The figure fully illustrates the process from the ERV (superfamily) to the LTR (order) level; other levels follow the same strategy and are represented by ellipses (...) for simplicity.

**Fig. S3.** Performance of the CNN module with different *k*-mer sizes. (A-D) Correspond to the results for accuracy, F1-score, precision, and recall, respectively. “*”,“**”, and “***” indicate significant differences with P < 0.05, P < 0.01, and P < 0.001, respectively.

**Fig. S4.** Performance of the RNN module with different both-end sequence extraction lengths. (A-D) Correspond to the results for accuracy, F1-score, precision, and recall, respectively. “*”,“**”, and “***” indicate significant differences with P < 0.05, P < 0.01, and P < 0.001, respectively.


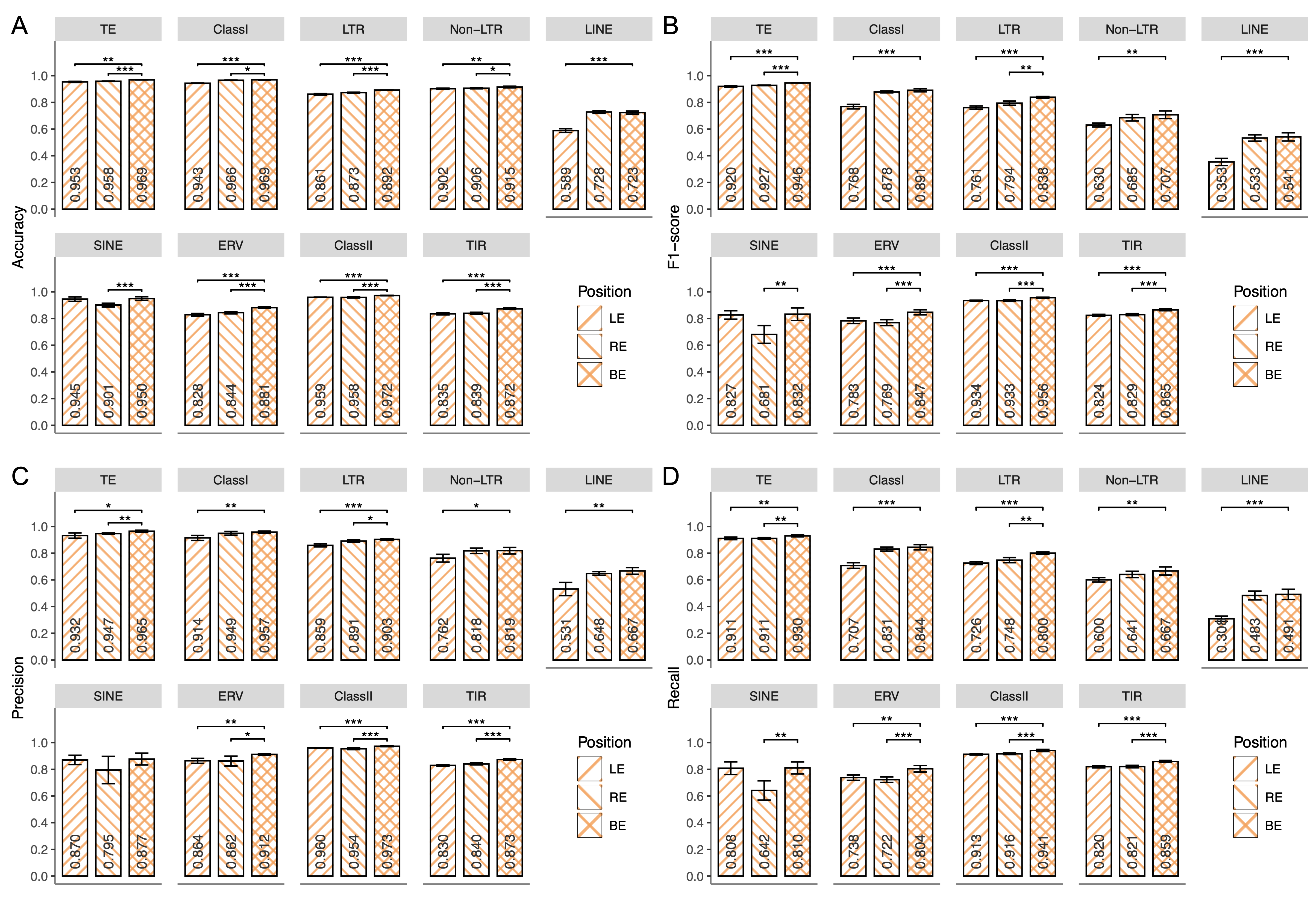


**Fig. S5.** Performance of different sequence feature extraction methods in the RNN model. LE: left end. RE: right end. BE: both ends. (A-D) Correspond to the results for accuracy, F1-score, precision, and recall, respectively. “*”,“**”, and “***” indicate significant differences with P < 0.05, P < 0.01, and P < 0.001, respectively.

**Fig. S6.** Performance of different alternative architectures of RNN module in CREATE. (A-E) Correspond to the results for MCC, accuracy, F1-score, precision, and recall, respectively. “*”,“**”, and “***” indicate significant differences with P < 0.05, P < 0.01, and P < 0.001, respectively.


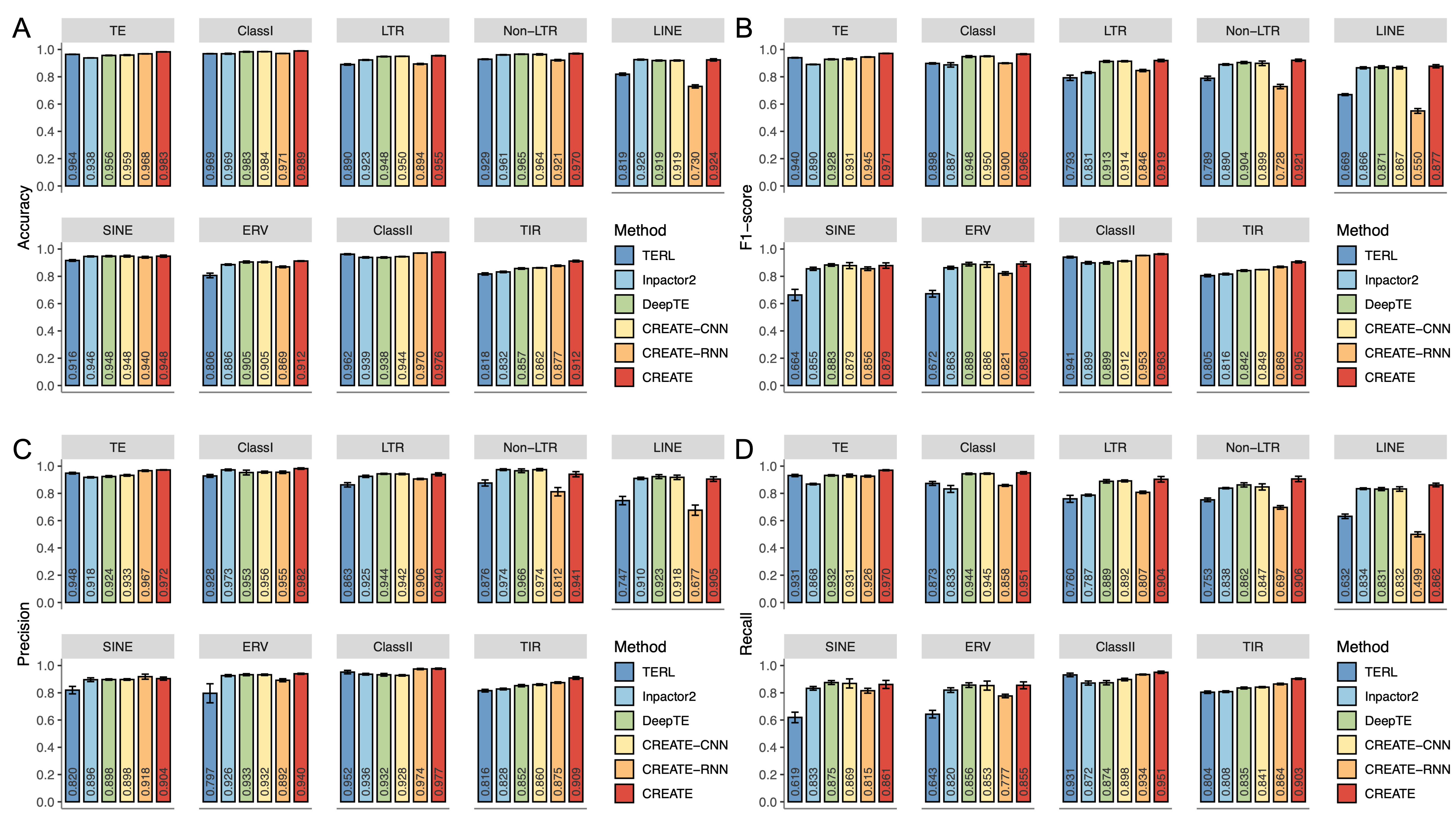


**Fig. S7.** Performance comparison of different methods on Dataset 1. (A-D) Correspond to the results of accuracy, F1-score, precision, and recall, respectively.


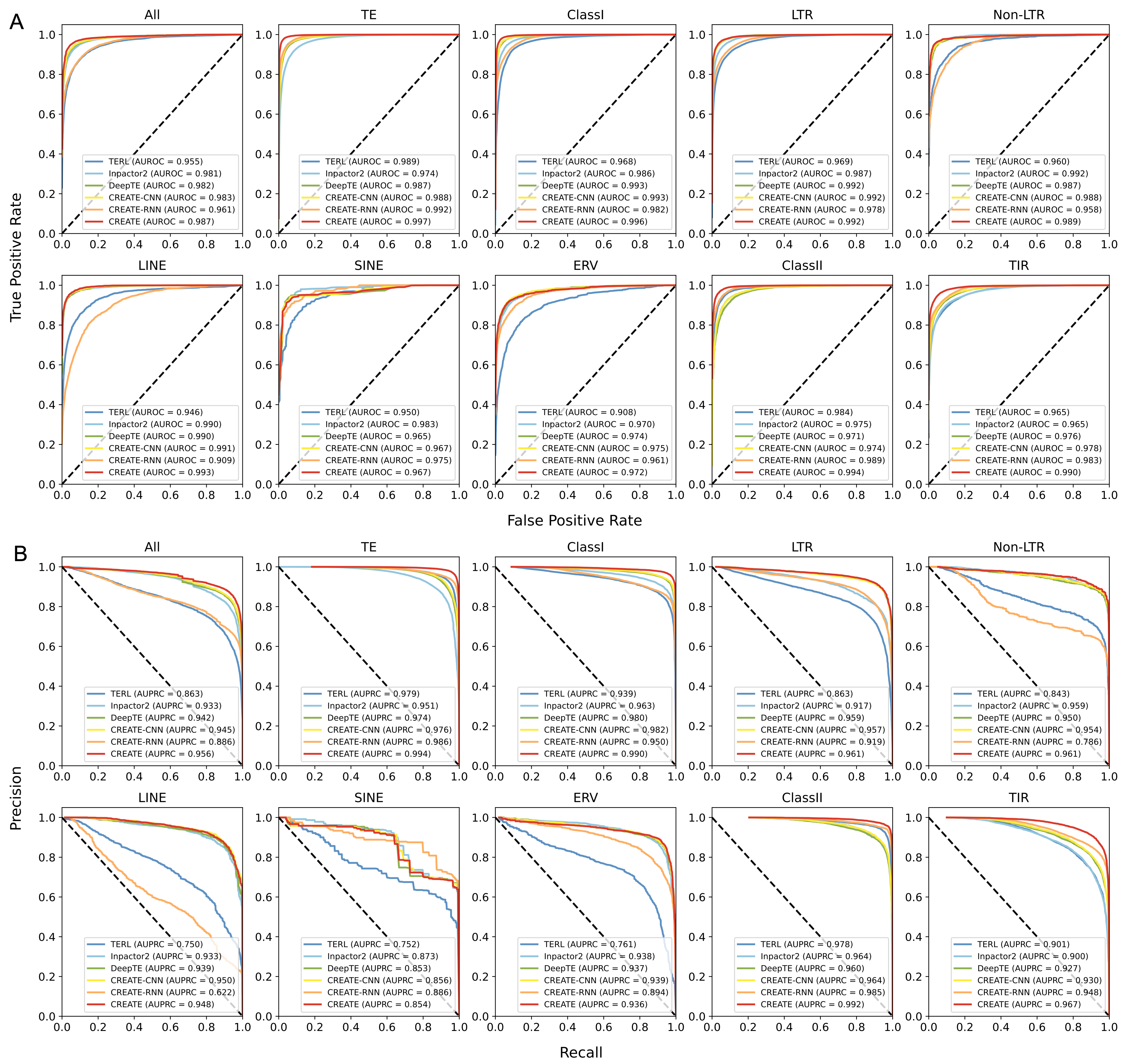


**Fig. S8.** Comparison of the receiver operating characteristic (ROC) and precision-recall (PR) curves of different methods on Dataset 1. For each model, the multiple classes are plotted as a single curve using macro-averaging.


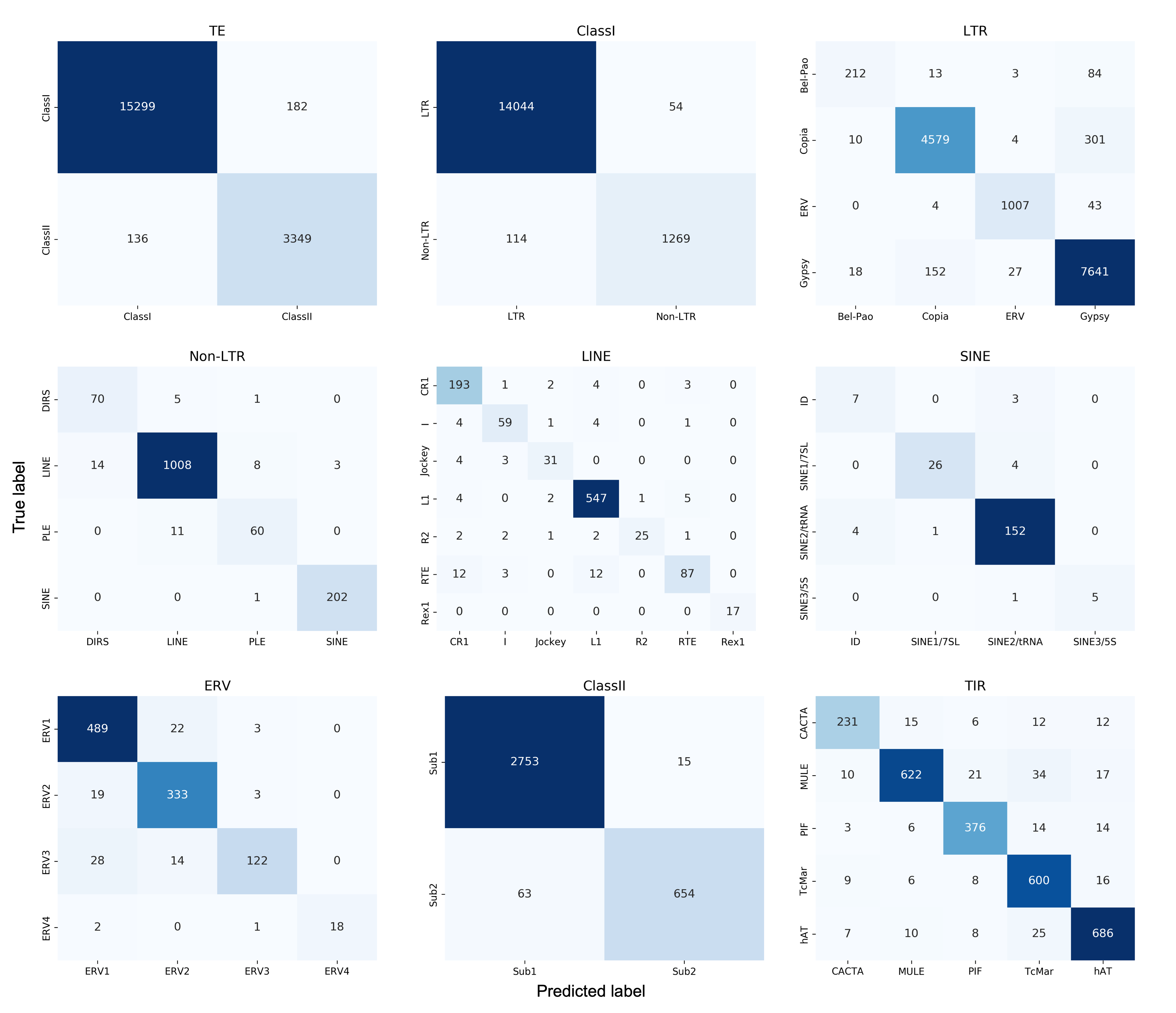


**Fig. S9.** Confusion matrices of the nine parent node classifiers on the test set of Dataset 1.


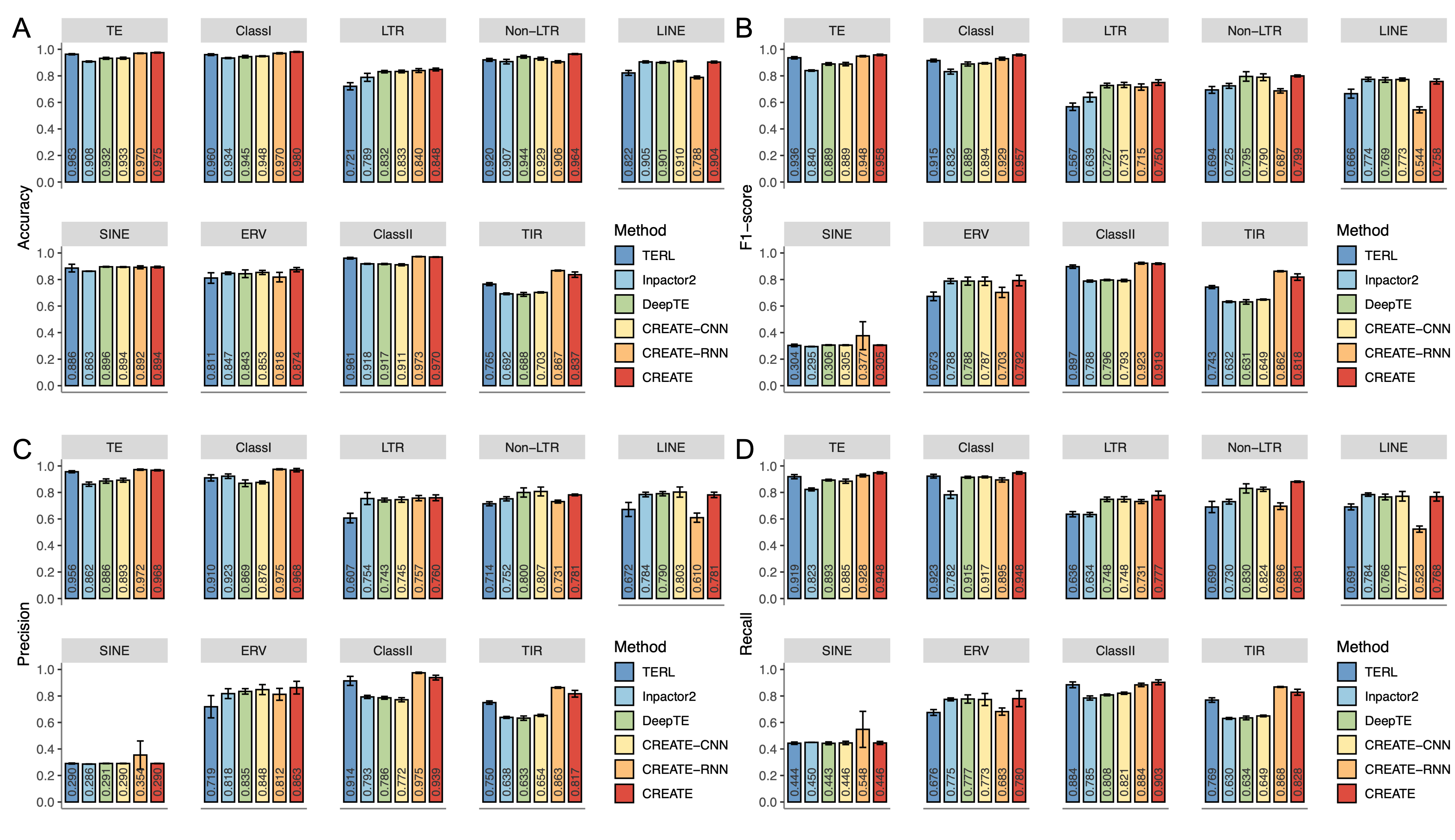


**Fig. S10.** Performance comparison of different methods on Dataset 2. (A-D) Correspond to the results of accuracy, F1-score, precision, and recall, respectively.


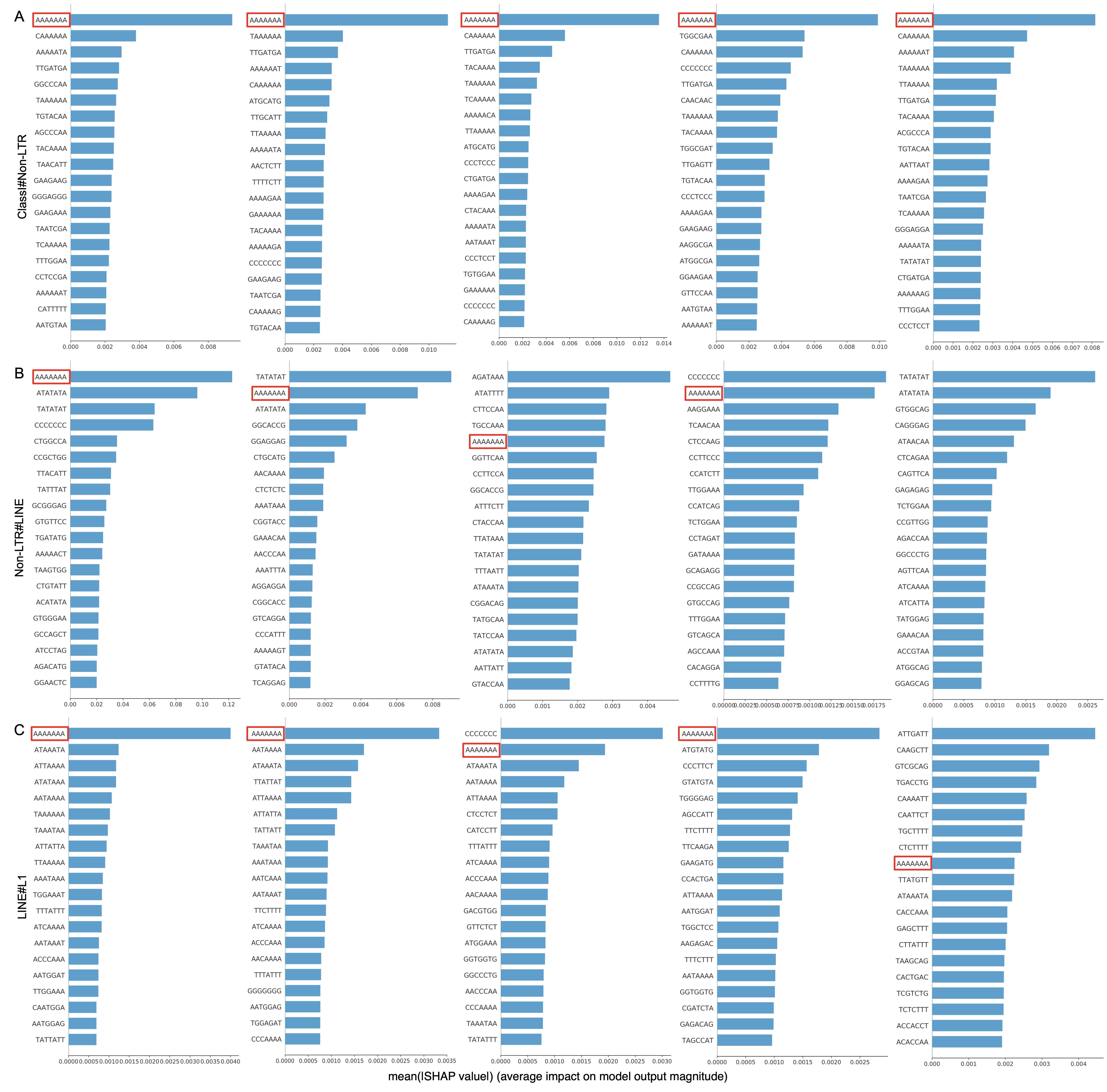


**Fig. S11.** SHAP-based 7-mer feature contributions in the CNN module. The top 20 most important features are shown. Each row contains five panels corresponding to five independent runs for robustness assessment. (A) Feature contributions in the Non-LTR prediction of the ClassI model. (B) Feature contributions in the LINE prediction of the Non-LTR model. (C) Feature contributions in the L1 prediction of the LINE model.


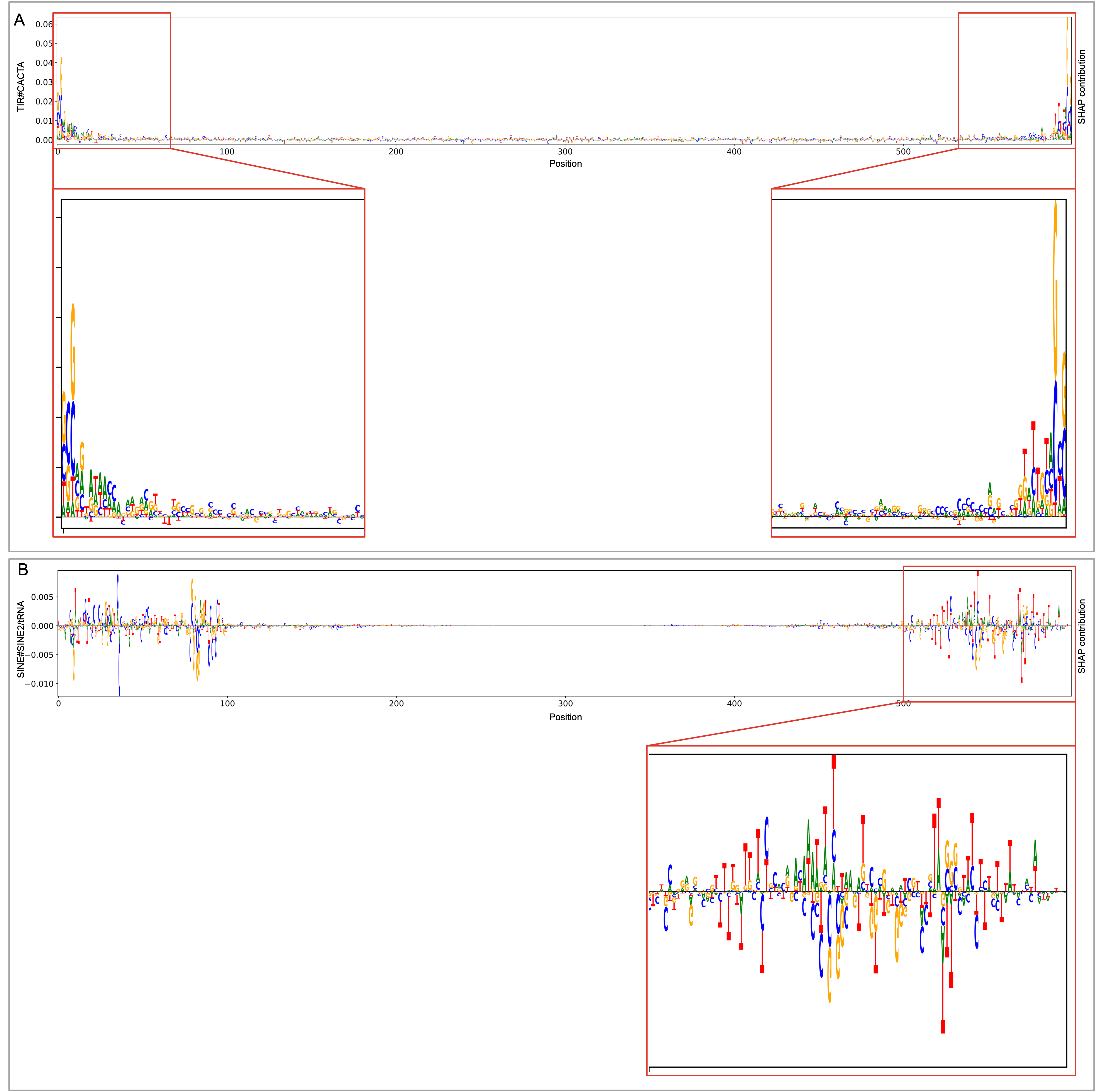


**Fig. S12.** SHAP-based sequence feature contributions in the RNN module. (A) CACTA elements of ClassII, showing pronounced terminal motifs relevant for subtype prediction. (B) SINE2/tRNA elements, showing T-rich motifs in the 3′ region. The near-zero contributions observed in the central regions are due to the relatively short length of SINE sequences, which range from 100 to 700 bp.


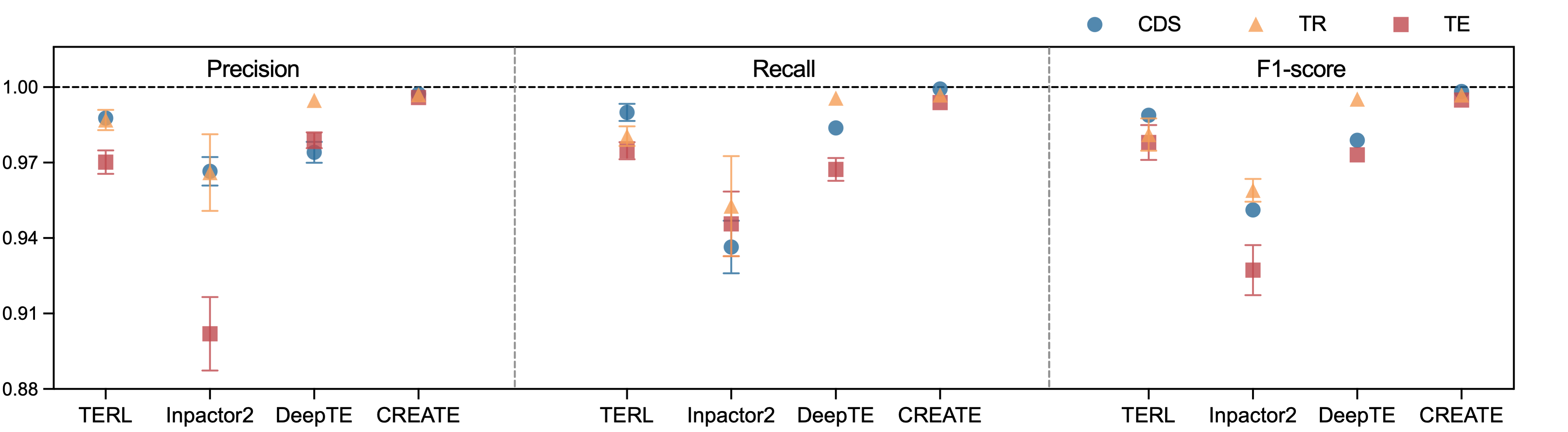


**Fig. S13.** Comparison of performance for distinguishing TEs and non-TEs. CDS, coding sequence; TR, tandem repeat.

**Supplementary Tables**

**Table S1.** Number of sequences in each TE group of Dataset 1. The table reports the number of TEs retained after preprocessing from each source database, along with the counts of sequences assigned to the training and test sets.

| **Class** | **Subclass** | **Order** | **Superfamily** | **Family** | **Database** | | | | | | | | | | | | | **Train** | | **Test** | **Total** | |  |
| --- | --- | --- | --- | --- | --- | --- | --- | --- | --- | --- | --- | --- | --- | --- | --- | --- | --- | --- | --- | --- | --- | --- | --- |
|  |  |  |  |  | **CicerSpTEdb** | **DPTEdb** | **Dfam** | **MnTEdb** | | **mips-REdat** | | **Repbase** | | **RepetDB** | | **SoyTEdb** | |  |  |  |  |  |  |
| ClassI | LTR | LTR | Bel-Pao |  | - | 260 | 235 | | - | | - | | 2,612 | | - | | - | | 2,795 | 312 | | 3,107 | |
|  |  |  | Copia |  | 2,674 | 3,542 | 46 | | 1,379 | | 10,128 | | 7,011 | | 10,761 | | 13,351 | | 43,998 | 4,894 | | 48,892 | |
|  |  |  | Gypsy |  | 814 | 7,181 | 896 | | 1,256 | | 17,419 | | 12,697 | | 19,167 | | 18,919 | | 70,511 | 7,838 | | 78,349 | |
|  |  |  | ERV | ERV1 | - | 243 | 2,645 | | - | | - | | 2,235 | | - | | - | | 4,609 | 514 | | 5,123 | |
|  |  |  |  | ERV2 | - | 280 | 1,709 | | - | | - | | 1,551 | | - | | - | | 3,185 | 355 | | 3,540 | |
|  |  |  |  | ERV3 | - | 9 | 810 | | - | | - | | 812 | | - | | - | | 1,467 | 164 | | 1,631 | |
|  |  |  |  | ERV4 | - | 9 | - | | - | | - | | 193 | | - | | - | | 181 | 21 | | 202 | |
|  | Non-LTR | DIRS |  |  | - | 42 | 51 | | - | | - | | 645 | | - | | 0 | | 662 | 76 | | 738 | |
|  |  | PLE |  |  | - | 2 | 168 | | - | | - | | 524 | | - | | - | | 623 | 71 | | 694 | |
|  |  | LINE | CR1 |  | - | 2 | 365 | | - | | - | | 1,647 | | - | | - | | 1,811 | 203 | | 2,014 | |
|  |  |  | I |  | - | 5 | 160 | | - | | - | | 517 | | - | | - | | 613 | 69 | | 682 | |
|  |  |  | Jockey |  | - | 1 | 91 | | - | | - | | 278 | | - | | - | | 332 | 38 | | 370 | |
|  |  |  | L1 |  | - | 1,300 | 1,899 | | 19 | | - | | 2,229 | | - | | 134 | | 5,022 | 559 | | 5,581 | |
|  |  |  | R2 |  | - | 1 | 15 | | - | | - | | 306 | | - | | - | | 289 | 33 | | 322 | |
|  |  |  | RTE |  | - | 9 | 397 | | 30 | | - | | 700 | | - | | - | | 1,022 | 114 | | 1,136 | |
|  |  |  | Rex1 |  | - | 1 | 28 | | - | | - | | 133 | | - | | - | | 145 | 17 | | 162 | |
|  |  | SINE | ID |  | - | - | 96 | | - | | - | | - | | - | | - | | 86 | 10 | | 96 | |
|  |  |  | SINE1/7SL |  | - | - | 255 | | - | | - | | 34 | | - | | - | | 259 | 30 | | 289 | |
|  |  |  | SINE2/tRNA |  | - | - | 885 | | - | | - | | 673 | | - | | - | | 1,401 | 157 | | 1,558 | |
|  |  |  | SINE3/5S |  | - | - | 11 | | - | | - | | 32 | | - | | - | | 37 | 6 | | 43 | |
| ClassII | Sub1 | TIR | CACTA |  | 853 | 57 | 106 | | 239 | | 464 | | 876 | | 68 | | 64 | | 2,451 | 276 | | 2,727 | |
|  |  |  | MULE |  | 2,300 | 144 | 29 | | 129 | | 210 | | 1,774 | | 117 | | 2,313 | | 6,312 | 704 | | 7,016 | |
|  |  |  | PIF |  | 116 | 93 | 124 | | 272 | | 141 | | 1,558 | | 210 | | 1,573 | | 3,674 | 413 | | 4,087 | |
|  |  |  | TcMar |  | 92 | 45 | 1,196 | | 1 | | 463 | | 2,790 | | 30 | | 1,743 | | 5,721 | 639 | | 6,360 | |
|  |  |  | hAT |  | 673 | 113 | 1,029 | | 1,041 | | 72 | | 4,004 | | 317 | | 65 | | 6,578 | 736 | | 7,314 | |
|  | Sub2 | Helitron |  |  | 437 | 4,048 | 420 | | 31 | | 4 | | 1,268 | | 845 | | 82 | | 6,418 | 717 | | 7,135 | |
| **Total** |  |  |  |  | **7,959** | **17,387** | **13,666** | | **4,397** | | **28,901** | | **47,099** | | **31,515** | | **38,244** | | **170,202** | **18,966** | | **189,168** | |

**Table S2.** Number of sequences in different TE groups for Datasets 2, 4, and 5. Dataset 2 includes sequences from both leaf and non-leaf nodes and is used to evaluate parent-node classification as well as hierarchical classification performance. Datasets 4 and 5 contain sequences with annotations at multiple hierarchical levels but are used exclusively to test hierarchical classification due to incomplete subcategory representation.

| **Class** | **Subclass** | **Order** | **Superfamily** | **Family** | **Dataset 2** | **Dataset 4** | **Dataset 5** |
| --- | --- | --- | --- | --- | --- | --- | --- |
| ClassI |  |  |  |  | - | - | - |
|  | LTR |  |  |  | 489 | 133 | 16 |
|  |  |  | Bel-Pao |  | 2,097 | - | - |
|  |  |  | Copia |  | 2,509 | 641 | 297 |
|  |  |  | Gypsy |  | 13,436 | 948 | 432 |
|  |  |  | ERV |  | 3 | - | - |
|  |  |  |  | ERV1 | 512 | - | - |
|  |  |  |  | ERV2 | 158 | - | - |
|  |  |  |  | ERV3 | 80 | - | - |
|  |  |  |  | ERV4 | 9 | - | - |
|  | Non-LTR |  |  |  | 295 | - | - |
|  |  | DIRS |  |  | 469 | - | - |
|  |  | PLE |  |  | 6 | - | - |
|  |  | LINE |  |  | - | 107 | 90 |
|  |  |  | CR1 |  | 254 | - | - |
|  |  |  | I |  | 51 | 213 | - |
|  |  |  | Jockey |  | 98 | 68 | - |
|  |  |  | L1 |  | 900 | 8 | - |
|  |  |  | R2 |  | 26 | 1 | - |
|  |  |  | RTE |  | 135 | - | - |
|  |  |  | Rex1 |  | 7 | - | - |
|  |  | SINE |  |  | 2 | 43 | 238 |
|  |  |  | ID |  | - | - | - |
|  |  |  | SINE1/7SL |  | 16 | - | - |
|  |  |  | SINE2/tRNA |  | 759 | - | - |
|  |  |  | SINE3/5S |  | 1 | - | - |
| ClassII |  |  |  |  | 219 | 9 | 4 |
|  | Sub1 | TIR |  |  | - | 63 | - |
|  |  |  | CACTA |  | 318 | 360 | 101 |
|  |  |  | MULE |  | 688 | 243 | 336 |
|  |  |  | PIF |  | 598 | - | - |
|  |  |  | TcMar |  | 1,185 | 342 | - |
|  |  |  | hAT |  | 1,577 | 34 | 238 |
|  | Sub2 | Helitron |  |  | 541 | 120 | 288 |
| **Total** |  |  |  |  | **27,438** | **3,333** | **2,040** |

**Table S3.** Number of sequences used for Dataset 3 and its corresponding training set. The training set comprises non-metazoan TIR sequences extracted from Dataset 1 training set.

| **Superfamily** | **Train** | **Test** |
| --- | --- | --- |
| CACTA | 2,368 | - |
| MULE | 6,877 | - |
| PIF | 3,426 | 5 |
| TcMar | 3,253 | 46 |
| hAT | 3,858 | 35 |
| **Total** | **19,782** | **86** |

**Table S4.** The number of CDS and TR sequences retained from the genomes of 21 selected species in Dataset 6. TE sequences for this three-class classification task were derived from the full Dataset 1 (Table S1). These sequences were used to evaluate the model’s performance in distinguishing TEs from non-TEs using 5-fold cross-validation.

| **Species No.** | **Species Name** | **Assembly Accession ID** | **CDS Count** | **TR Count** |
| --- | --- | --- | --- | --- |
| 1 | Aedes aegypti | GCF_002204515.2 | 5,650 | 26,756 |
| 2 | Arabidopsis lyrata | GCF_000004255.2 | 11,131 | 1,523 |
| 3 | Arabidopsis thaliana | GCF_000001735.4 | 3,862 | 897 |
| 4 | Cannabis sativa | GCF_029168945.1 | 10,612 | 13,917 |
| 5 | Carica papaya | GCF_000150535.2 | 7,120 | 4,917 |
| 6 | Cicer arietinum | GCF_000331145.1 | 9,477 | 10,750 |
| 7 | Danio rerio | GCF_000002035.6 | 9,741 | 62,012 |
| 8 | Glycine max | GCF_000004515.6 | 16,523 | 12,367 |
| 9 | Gossypium raimondii | GCF_025698545.1 | 14,121 | 12,330 |
| 10 | Malus domestica | GCF_002114115.1 | 12,910 | 3,078 |
| 11 | Medicago truncatula | GCF_003473485.1 | 11,500 | 3,192 |
| 12 | Morus notabilis | GCF_000414095.1 | 8,045 | 6,960 |
| 13 | Oryza sativa Japonica Group | GCF_034140825.1 | 6,986 | 7,904 |
| 14 | Phoenix dactylifera | GCF_009389715.1 | 9,844 | 9,995 |
| 15 | Populus trichocarpa | GCF_000002775.5 | 11,785 | 4,437 |
| 16 | Rosa chinensis | GCF_002994745.2 | 12,526 | 4,420 |
| 17 | Sitophilus oryzae | GCF_002938485.1 | 6,172 | 5,238 |
| 18 | Solanum tuberosum | GCF_000226075.1 | 11,994 | 2,331 |
| 19 | Sorghum bicolor | GCF_000003195.3 | 6,898 | 4,096 |
| 20 | Vitis vinifera | GCF_030704535.1 | 10,207 | 5,311 |
| 21 | Zea mays | GCF_902167145.1 | 9,405 | 8,266 |
| **Total** |  |  | **206,509** | **210,697** |

**Table S5.** Comparison of CNN configurations across five representative parent nodes*****. Configurations vary in convolutional layer number, kernel size, kernel number per layer, and fully connected layer number. Performance metrics are reported as mean ± SD from 5-fold cross-validation on the training sets. The final selected configuration is highlighted in bold. CONV: convolutional layer; FC: fully connected layer.

| **CNN architecture** | | | | | **Performance (MCC)** | | | | |
| --- | --- | --- | --- | --- | --- | --- | --- | --- | --- |
| CONV layers | Kernel size | Kernel per  CONV layer | FC layers | Neurons per  FC layer | LTR | LINE | SINE | ClassII | TIR |
| **3** | **1×3** | **64–128–256** | **1** | **128** | **0.904±0.003** | **0.833±0.013** | **0.891±0.029** | **0.809±0.009** | **0.809±0.004** |
| 2 | 1×3 | 64–128 | 1 | 128 | 0.900±0.005 | 0.828±0.013 | 0.878±0.011 | 0.811±0.010 | 0.807±0.006 |
| 4 | 1×3 | 32–64–128–256 | 1 | 128 | 0.899±0.004 | 0.820±0.011 | 0.867±0.026 | 0.811±0.006 | 0.799±0.006 |
| 3 | 1×3 | 32–64–128 | 1 | 128 | 0.903±0.005 | 0.832±0.009 | 0.877±0.013 | 0.805±0.013 | 0.812±0.011 |
| 3 | 1×5 | 64–128–256 | 1 | 128 | 0.904±0.005 | 0.828±0.022 | 0.866±0.029 | 0.810±0.008 | 0.801±0.007 |
| 3 | 1×3 | 64–128–256 | 2 | 256–128 | 0.904±0.004 | 0.833±0.023 | 0.889±0.026 | 0.806±0.010 | 0.805±0.008 |

* Since the hierarchical framework includes nine parent nodes, we selected five representative nodes for detailed model comparison: LTR, LINE, SINE, ClassII, and TIR. For retrotransposons, we selected three parent nodes based on their distinct characteristics. LTR elements are widely distributed across genomes, represent the most abundant category in our integrated datasets, and play important roles in genome evolution and regulation; LINEs have multiple subclasses (seven) with strong class imbalance (rare subtype account for only 1.65% of all LINE sequences), making them a challenging test case; SINEs, short elements typically 100–700 base pairs long, have distinct structural features that make them particularly informative for accurate classification and for exploring lineage-specific genomic evolution and regulatory roles. In addition, for DNA transposons, we selected the ClassII parent node, which represents classification at the order level rather than the superfamily level, providing a broader view of TE diversity; we also selected TIR as representative Class II elements, whose characteristic terminal inverted repeats make them particularly informative for studying DNA transposon structure. This selection allowed us to cover a range of data characteristics and biological contexts while keeping the experimental comparisons feasible and meaningful.

**Table S6.** Comparison of RNN architectures across five representative parent nodes. Configurations vary in GRU layer depth and neuron numbers per layer. Performance metrics are reported as mean ± SD from 5-fold cross-validation on the training sets. The final selected configuration is highlighted in bold. Results that outperform the selected RNN model by 0.005 are marked with an underline for clarity.

| **RNN architecture** | | | | **Performance (MCC)** | | | | |
| --- | --- | --- | --- | --- | --- | --- | --- | --- |
| GRU layers | Neurons per  GRU layer | FC layers | Neurons per  FC layer | LTR | LINE | SINE | ClassII | TIR |
| **2** | **128–64** | **1** | **128** | **0.807±0.001** | **0.553±0.018** | **0.858±0.037** | **0.914±0.006** | **0.836±0.008** |
| 1 | 128 | 1 | 128 | 0.791±0.005 | 0.527±0.012 | 0.853±0.036 | 0.909±0.008 | 0.827±0.006 |
| 3 | 128–64–32 | 1 | 128 | 0.806±0.003 | 0.565±0.011† | 0.847±0.036 | 0.915±0.005 | 0.836±0.005 |
| 2 | 256–128 | 1 | 128 | 0.808±0.006 | 0.532±0.017 | 0.852±0.055 | 0.910±0.008 | 0.833±0.006 |

† For LINE, GRU(128–64–32)+FC(128) outperforms GRU(128–64)+FC(128), but the RNN module alone (MCC 0.527–0.565) underperforms compared with the CNN model (MCC 0.833). For SINE, GRU(128–64)+FC(128) improves performance by 0.011 over GRU(128–64–32)+FC(128). GRU(128–64–32)+FC(128) increases average training time per fold by ~36% for LTR (33.95 vs. 24.87 min). Considering both performance and computational efficiency, GRU(128–64)+FC(128) was selected as the final architecture.

**Table S7.** Detailed network configurations of the baseline deep learning methods used for comparison. CONV: convolutional layer; POOL: pooling layer; FC: fully connected layer.

|  | **TERL** | **Inpactor2** | **DeepTE** |
| --- | --- | --- | --- |
| Network Type | CNN | FNN | CNN |
| CONV Layers | 3 layers | - | 3 layers |
| - Number of Filters | 64, 32, 32 | - | 100, 150, 225 |
| - Kernel Size | 6×20, 1×20, 1×35 | - | 1×3, 1×3, 1×3 |
| - Stride | 1, 1, 1 | - | 1, 1, 1 |
| POOL Layers | Average pooling† | - | Max pooling† |
| - Kernel Size | 1×10, 1×15, 1×15 | - | 1×2, 1×2, 1×2 |
| - Stride | 1×10, 1×15, 1×15 | - | 1×2, 1×2, 1×2 |
| FC Layers | 2 layers | 3 layers | 1 layer |
| - Neurons per Layer | 1000, 500 | 200, 200, 200 | 128 |
| Learning Rate | 0.001 | 0.001 | 0.001 |
| Optimizer | Adam | Adam | Adam |
| Batch Size | 32 | 128 | 32 |
| Number of Epochs | 10 | 200‡ | 10 |

† POOL layers are applied immediately after each CONV layer.

‡ Training employed early stopping, halting after 10 consecutive epochs without improvement in validation performance. 10% of the training set was randomly selected as the validation set.
